# Peak alpha frequency is associated with pain severity in Long COVID patients with new-onset chronic pain

**DOI:** 10.3389/fpain.2026.1893610

**Published:** 2026-07-29

**Authors:** Bárbara Silva-Passadouro, Omar Khoja, Alexander J. Casson, Ioannis Delis, Christopher Brown, Manoj Sivan

**Affiliations:** 1 Leeds Institute of Rheumatic and Musculoskeletal Medicine, School of Medicine, University of Leeds, Leeds, United Kingdom; 2 Physiotherapy Department, King Salman Bin Abdulaziz Medical City, Madinah Health Cluster, Madinah, Saudi Arabia; 3 Department of Electrical and Electronic Engineering, University of Manchester, Manchester, United Kingdom; 4 School of Biomedical Sciences, Faculty of Biological Sciences, University of Leeds, Leeds, United Kingdom; 5 School of Electrical and Computer Engineering, National Technical University of Athens, Athens, Greece; 6 Department of Psychology, Institute of Population Health, University of Liverpool, Liverpool, United Kingdom; 7 National Demonstration Centre in Rehabilitation Medicine, Leeds Teaching Hospitals NHS Trust, Leeds, United Kingdom

**Keywords:** electroencephalography, pain, peak alpha frequency, post acute covid syndrome, post-COVID syndrome, resting-state

## Abstract

**Background:**

New-onset chronic pain is a common and debilitating symptom of Long COVID (LC) that remains not fully understood in terms of pathophysiology and therapeutic targets. A growing body of evidence in chronic pain syndromes similar to LC demonstrates an association between EEG alpha oscillatory activity and the experience of pain, with clinical studies showing maladaptive changes, particularly a slowing of alpha activity.

**Aims:**

This study aims to investigate the association between EEG alpha oscillatory activity and pain perception in new-onset LC-chronic pain.

**Methods:**

We recruited 31 individuals (20 females) with a clinical diagnosis of LC reporting new-onset chronic pain and 31 healthy pain-free age- and sex-matched controls. Participants completed questionnaires regarding symptoms and psychological functioning prior to recording eyes-open resting-state EEG. Peak alpha frequency (PAF) and alpha band (8–13 Hz) spectral power were extracted from EEG signals.

**Results:**

Lower PAF over the posterior scalp region was significantly associated with higher LC-chronic pain severity when controlling for age, depression and the use of centrally acting medication. This finding was consistent across PAF estimation methods. No differences in PAF were observed between the LC patient group and the healthy pain-free control group. Alpha power did not differ between groups and was not associated with pain severity.

**Discussion:**

Together, these findings suggest that PAF may index individual differences in pain severity within LC-associated chronic pain, although the absence of overall patient-control differences indicates that PAF should not be interpreted as a disease-specific biomarker and warrants further longitudinal research.

## Introduction

Pain is a common and often debilitating symptom of Long COVID (LC), a multisystem post-infection syndrome experienced by over 400 million people globally (1). New-onset musculoskeletal LC-chronic pain refers to pain of musculoskeletal origin that persists or arises following resolution of the acute phase of coronavirus disease-19 (COVID-19) infection and lasts longer than three months (2). Chronic pain is consistently cited as one of the most commonly reported symptoms of LC, estimated to be present in at least 10 to 20% of LC cases (3–7), with prevalence rates going up to 50% in patients hospitalised due to COVID-19 illness (8–10).

The widespread distribution of pain typically observed in LC and its comorbid association with other central nervous system-related symptoms such as sleep disturbances, depression, fatigue and cognitive impairments, are suggestive of a nociplastic-type pain (11–16). In fact, a significant proportion of LC patients fulfil the diagnostic criteria for Fibromyalgia Syndrome (FMS) (17–20), where nociplastic pain is typically present (21). The lack of reliable diagnostic tools and effective biomarkers makes nociplastic pain difficult to identify and treat, which has important clinical implications for LC, where the coexistence of mixed pain types adds further complexity to clinical management.

Emerging evidence suggests that neurophysiological markers, such as oscillatory cortical neural activity measured via electroencephalography (EEG), may offer valuable insights into the neural basis of chronic pain. There has been particular interest directed toward changes in alpha oscillatory activity (8–13 Hz), the dominant rhythm in relaxed wakeful EEG prominently observed over sensory regions (22), as a potential biomarker of chronic pain (23, 24). For instance, studies have reported a slowing of EEG alpha activity, as indexed by decreased peak alpha frequency (PAF), in individuals with chronic pain, including those with FMS, compared to pain-free controls (25–27). PAF exhibits high and relatively stable between-subject variability that has been associated with individual differences in cognitive functioning and sensory processing (28, 29). It is thought to reflect underlying thalamocortical network dynamics and has been functionally linked to the temporal resolution of sensory processing, as well as the gating of incoming sensory information (30), including nociceptive signals. PAF slowing has been associated with heightened pain sensitivity and sustained pain in some experimental models (31, 32), with emerging evidence also suggesting a link between pain-free pre-surgical PAF and post-operative pain severity (33, 34). Beyond PAF, reduced alpha power has also been observed in FMS patients (35–37), although this finding does not appear to be fully consistent with observations in conditions associated with neuropathic pain (26, 38, 39). Given the overlap in clinical features, it may be hypothesised that LC-chronic pain, which phenotypically resembles FMS, is associated with alterations in EEG alpha activity comparable to those observed in FMS (40).

This study aims to investigate EEG alpha oscillatory features of new-onset LC chronic pain. The data was derived from the Musculoskeletal Pain in Long COVID (MUSLOC) research project (clinicaltrials.gov NCT05358119), a prospective longitudinal study aiming to investigate the characteristics and mechanisms underlying LC-chronic pain in a sample of LC patients with new-onset musculoskeletal chronic pain. Here, we present the findings of cross-sectional analysis of resting-state EEG data from the same cohort. A healthy pain-free control group was recruited separately for comparative analysis with patient data. We hypothesised that (a) lower PAF is associated with higher severity of new-onset LC chronic pain; (b) LC patients with new-onset chronic pain have lower PAF compared to pain-free controls; and (c) alpha band power is decreased in patients compared to controls.

## Methods

### Participants

Thirty-one adult patients with LC and new-onset chronic pain were recruited primarily from the Leeds Long COVID Community Rehabilitation Service, as well as through social media and patient support groups. Following initial interest, individuals were contacted to confirm eligibility and to explain the study aims and procedures. The criteria for inclusion in the study was: (a) aged 18 years or older; (b) tested positive for COVID-19 or reported COVID-19 symptoms that were confirmed by an independent clinician; (c) had a clinical diagnosis of LC as per the NICE guidelines definition (41); (d) presented with new-onset musculoskeletal chronic pain as defined by the ICD-11 criteria (2) since COVID-19 infection. Potential participants were excluded if they presented with chronic pain prior to COVID-19 infection; were unable to read and understand English; had history of seizures or clinical diagnosis of epilepsy; or were unable to comply with study procedures.

The control group consisted of 31 sex- and age-matched (± 3 years) healthy volunteers with no clinical history of seizures or epilepsy and not experiencing any pain. Healthy controls were recruited in a separate study (outside the MUSLOC study for patients) via social media advertisement and university mailings lists. The EEG equipment, recording environment, recording parameters, protocol, signal preprocessing and EEG feature extraction procedure were identical for patient group and healthy controls.

All participants provided written informed consent prior to any study procedure and were informed of their right to withdraw from the study at any time. Participation in the study was entirely voluntary. The MUSLOC project was registered on clinicaltrials.gov (NCT05358119) and approved by the London—Central Research Ethics Committee (REC), the Health Research Authority (HRA) and Health and Care Research Wales (HCRW) (Ref 21/PR/1377). Healthy participants were recruited under ethical approval from the University of Leeds' Faculty of Biological Sciences ethics committee (BIOSCI 23-008). All procedures were conducted in accordance with the Declaration of Helsinki.

### Questionnaires

Patients completed the self-assessment questionnaires listed below on the same day as EEG data collection:

(a) Demographic information form: to collect demographic data, including items in regard to participant's age, sex, ethnicity, marital status, weight, height, pre-existing medical conditions, smoking status, COVID-19 related information (i.e., COVID-19 infection and vaccination history, hospitalisation due to COVID-19), employment category and status following COVID-19 pandemic and LC.
(b) COVID-19 Yorkshire Rehabilitation Scale (C19-YRS): the first validated scale developed specifically for assessment and monitoring of patients with LC and used in LC clinics across the UK (42). It consists of 15 items relative to severity of core symptoms of LC (including breathlessness, cough, swallowing, fatigue, continence, pain, cognition, anxiety, depression, post-traumatic stress disorder) and impact on daily functions (including communication, mobility, personal care, other activities of daily living, social role). Each item is scored on two numeric rating scales ranging from 0 to 10 relative to their symptoms pre- and post-COVID respectively. Items 1–10 capture symptom severity (scores 0–100) and items 11–15 capture functional disability (scores 0–50). The C19-YRS also measures the severity of other common LC symptoms (including palpitations, dizziness, weakness, sleep problems, fever, skin rash) and overall health (scores 0–10; score of 0 for worst health and 10 for best health imagined). The post-COVID pain item of the C19-YRS was used as pain severity score (range: 0–10) in the analysis for this study.
(c) Patient Health Questionnaire 9 (PHQ-9): a screening, diagnostic and monitoring tool for depression based on the diagnostic criteria set by the Diagnostic and Statistical Manual of Mental Disorders (43). It consists of nine items rated on a 4-category response scale (0—Not at all; 1—Several days; 2—More than half the days; 3—Nearly every day) that ask individuals to rate how often they have been bothered by symptoms of depression in the past two weeks. The level of severity of depression is assessed using the total scores (range: 0–27), with 10 being the standard cut-off score for major depression (44).

Healthy volunteers completed a demographic information form and the PHQ-9.

### EEG recordings and data acquisition

Resting-state EEG was selected as we were interested in the intrinsic brain oscillatory activity associated with the persistent ongoing pain state in LC, that is experienced independent of movement or external nociceptive stimuli. EEG signals were collected using a BrainVision actiCHamp Plus amplifier system (Brain Products GmbH, Gilching, Germany) with active Ag/AgCl electrodes attached to a 64-channel ActiCAP (Brain Products GmbH, Wörthsee, Germany) positioned according to the 10–20 international electrode placement system. Participants were sat comfortably on a chair in a dim light room. They were instructed to remain relaxed and keep their eyes open whilst fixating on a cross on the wall approximately one meter from the chair. Eyes-open was chosen over eyes-closed to help reduce drowsiness and mitigate risk of falling asleep. Two blocks of six minutes each of resting-state EEG signal were recorded for each participant. EEG signals were recorded at a sampling rate of 1,000 Hz and electrode impedances were kept at 10 kΩ or below. All EEG electrodes were referenced to the FCz electrode. The ground electrode was placed at Fpz channel position.

### EEG preprocessing

Pre-processing and cleaning of raw EEG signals was implemented in MATLAB (ver. R2023b) custom scripts using the EEGLAB toolbox (45). Data were filtered using zero-phase, linear phase finite impulse response (FIR) filters. A high-pass filter and low-pass filter were applied sequentially at frequencies 0.5 Hz and 35 Hz respectively, followed by attenuation of 50 Hz power line interference by notch filtering. After downsampling to 250 Hz, EEG data recorded from each participant were merged, resulting in one single 12 min dataset per participant. Flat or excessively noisy channels were then removed, with a mean of 0.4 channels per participant (range: 0–4). The Artifact Subspace Reconstruction (ASR) algorithm was applied to automatically correct bad data periods with variance larger than the rejection threshold of 30 standard deviations (*k* parameter) relative to a clean portion of EEG signals (*clean_rawdata* plugin for EEGLAB) (46). Other ASR parameters were set to default. The data were then re-referenced to the common average. Independent component analysis (ICA) was computed using EEGLAB function *runica* (47), with the number of components limited to the effective data rank (45); data were internally whitened using a principal component analysis (PCA)-based step prior to ICA optimisation (47, 48). Components generated by eye blinks, saccades, muscle activity and heart artifacts were identified with the support of ICLabel classification (49), with any components labelled as >90% non-brain artifact automatically flagged for rejection. Further rejection was performed manually through visual inspection based on component topographies, frequency distribution and time courses (median of total number of ICA components rejected = 9, range: 2–19, IQR: 6.25–11). Rejected channels were interpolated after ICA cleaning.

### EEG feature extraction

Power spectral density for each channel was estimated from the cleaned EEG signals using the Welch's method (2 s segments, no overlap, Hanning window). The absolute spectral power of the alpha frequency band (8–13 Hz) was extracted using the trapezoidal integration method of estimation of the area under the spectral curve (MATLAB function *trapz*). The power of other canonical frequency bands was also extracted for exploratory supplementary analysis, with ranges set as follows: delta (2–4 Hz), theta (4–8 Hz), and beta (13–30 Hz).

Peak alpha frequency was derived using two different methods. Max-PAF was estimated as the frequency in the alpha range (8–13 Hz) that exhibits maximum Welch's power spectral density. The maximum Welch's power spectral density was also extracted and set as max-PAF power spectral density. The second method used the spectral parameterisation algorithm [frequency range = [1, 30] Hz; default hyperparameters: peak width limits = [1, 8] Hz, maximum number of peaks = 6, minimum peak height = 0.15 arbitrary units, peak threshold = 2 standard deviations] to decompose Welch's power spectral data into periodic and aperiodic components (*specparam* toolbox, formerly *fooof*, version 1.1.0; Python wrapper version 1.1.0) (50). This approach accounts for the contribution of aperiodic EEG activity, characterised by a 1/f-like distribution, which co-occurs with periodic activity and can confound the estimation of true oscillatory peaks within canonical frequency bands. For instance, changes or interindividual variation in PAF or power at a specific frequency band can be conflated with differences in aperiodic activity, thus leading to misinterpretation of underlying physiological phenomena (51). The specparam algorithm iteratively fits Gaussian curves to model each oscillatory peak in the aperiodic-adjusted power spectrum, derived by subtracting the estimated 1/f aperiodic component. The algorithm outputs parameters that define the aperiodic component (exponent and offset), the putative periodic component (centre frequency, power and bandwidth for each fitted Gaussian) and overall model fit (*R*^2^ and error). For the present study, aperiodic-adjusted PAF was set as the frequency with the strongest Gaussian peak identified within the alpha range. Power spectral density of the aperiodic-adjusted PAF was also extracted from model outputs. Channels for which spectral parameterisation did not detect an alpha oscillatory peak were identified for each participant and excluded from max-PAF estimation for that participant (mean of 3.4 channels per participant, range: 0–24). We excluded channels with specparam model fit below <0.9 *R*^2^ from analysis as suggested by Gyurkovics et al. (52).

Peak alpha frequency and power estimates were median averaged across respective channels to extract global (all electrodes) and regional PAF for each participant. For regional analysis, three regions of interest were defined pragmatically to capture coarse topographical variation in alpha-band features without imposing a strong *a priori* regional hypotheses: anterior region (comprising electrodes Fp1, Fp2, AF7, AF3, AFz, AF4, AF8, F7, F5, F3, F1, Fz, F2, F4, F6 and F8); central region (comprising electrodes FT9, FT7, FC5, FC3, FC1, FC2, FC4, FC6, FT8, FT10, T7, C5, C3, C1, Cz, C2, C4, C6, T8, TP9, TP7, CP5, CP3, CP1, CPz, CP2, CP4, CP6, TP8 and TP10); and posterior region (comprising electrodes P7, P5, P3, P1, Pz, P2, P4, P6, P8, PO7, PO3, POz, PO4, PO8, O1, Oz and O2).

### Statistical analysis

Statistical analysis was conducted in RStudio (version 2024.12.1). Data are presented as mean ± standard deviation unless otherwise indicated. Statistical significance for all tests was set at *α* = 0.05. Participants with missing or incomplete questionnaires were excluded from the analysis relative to that specific questionnaire.

Spearman's rank correlation was calculated between max-PAF and aperiodic-adjusted PAF estimates derived from each region to assess the robustness and consistency of the estimates across PAF extraction methods.

#### Primary aim 1. Association between PAF and pain severity in LC patients with new-onset chronic pain

The association between pain symptoms and EEG PAF was investigated using multiple linear regression analysis. Peak alpha frequency derived from the two estimation methods (max-PAF and aperiodic-adjusted PAF) was entered as a predictor in separate models, with C19-YRS pain severity subscale scores as dependent variable. Global PAF estimates and estimates derived from each region (anterior, central and posterior) were tested separately. Depression and older age have been linked with decreased PAF (28, 53–55). Hence, we adjusted for the potential confounding effects of age and depression (as self-reported in the PHQ-9) in our analysis. All model assumptions were examined through model diagnostic plots including residual plots and Q-Q plots. The models were specified as:

Painseverityscore∼PAF+age+depressionscore

*P* values from the core adjusted models (derived from the formula above) were corrected for multiple testing using false discovery rate (FDR) correction. FDR correction was applied across models derived from global and all regional estimates, separately for each family of models relevant to the different PAF estimation methods. For transparency, we also present the outputs of simple regression models (i.e., *Pain severity score ∼ PAF*) and models including each of the confounding variables individually to show the effects of introducing each to the adjusted model in Supplementary materials.

Significant PAF-pain associations were followed up with further analysis to assess the robustness of the findings. A sensitivity analysis was conducted to determine whether the use of centrally acting medication (i.e., opioids, gabapentinoids, SSRIs, tricyclic antidepressants) confounded the observed associations, by adding medication status as a binary factor in the model (taking centrally acting medication = 1, not taking centrally acting medication = 0). The symptom-specificity of the findings was assessed by including the listed outcomes as dependent variables in separate models: severity of other neurological symptoms of LC (i.e., fatigue and cognitive impairment subscales of the C19-YRS), overall LC symptom severity, duration of pain symptoms and pain widespreadness (i.e., painful sites were identified during clinical examination and recorded on a pain body map, with total number of painful sites calculated from the marked body regions). Additional exploratory analysis was performed with power of the strongest peak within the alpha range (PAF power spectral density) as independent variable, to test whether PAF power spectral density independently predicts pain.

#### Primary aim 2. Comparative analysis of PAF between LC patient and healthy pain-free control groups

All variables were tested for normal distribution (using Shapiro–Wilk test, Q-Q plots and histograms) and equality of variances (using Levene's test) prior to between-groups analysis. If found non-normally distributed, the channel-averaged EEG feature values were transformed using logarithmic transformation (log10).

Between-group differences in PAF were tested with multiple linear regression models including a 2-level group factor (patients vs. controls). Peak alpha frequency derived from the two estimation methods (max-PAF and aperiodic-adjusted PAF) was entered as dependent variable in separate models. Additionally, global PAF estimates and estimates derived from each region (anterior, central and posterior) were tested separately. The models were specified as:

PAF∼group+age

#### Primary aim 3. Comparative analysis of alpha band power between LC patient and healthy pain-free control groups

Comparative analysis of alpha band power between groups followed the methods described for primary aim 2, but with alpha power estimates as dependent variable.

#### Additional exploratory analysis (Supplementary materials)

In addition to the prespecified primary analyses, we conducted a series of exploratory and confirmatory analyses to further test EEG alpha oscillatory activity patterns associated in LC-chronic pain. This included: (a) exploratory subgroup-based analysis by stratifying patients based on pain severity into moderate and severe pain subgroups using a median split; (b) sensitivity analysis of group differences in PAF accounting for the potential confounding effects of depression; (c) exploratory analysis of the association between absolute spectral power of other canonical EEG frequency bands (delta, theta and beta) and pain severity within patient group; and (d) exploratory comparative analysis of absolute spectral power of other canonical EEG frequency bands between LC patients and controls. No adjustments were made for multiple comparisons given the exploratory nature of these analyses. All methods and results related to these analyses are reported in the Supplementary materials.

## Results

### Demographics and symptom severity

A summary of participant demographics for both the LC patient group and the healthy pain-free control group can be found in Table 1. Table 2 shows a summary of clinical characteristics and severity of neurological and psychological symptoms of LC for the patient group. A full list of all the symptoms reported on the C19-YRS, including pre- and post-COVID scores, can be found in Supplementary Table S1.

**Table 1:** Summary of demographic and psychological variables for the patient and healthy pain-free control groups.

Variables	Long COVID patient group (*n* = 31)	Healthy pain-free control group (*n* = 31)
Demographic characteristics		
Age, mean (SD), years	46.4 (11.1)	46.6 (11.0)
Sex, *n* (%)		
Female	20 (64.5)	20 (64.5)
Male	11 (35.5)	11 (35.5)
Ethnicity, *n* (%)		
White	26 (83.9)	23 (74.2)
Asian	5 (16.1)	7 (22.6)
Black	0 (0.0)	0.0 (0.0)
Mixed	0 (0.0)	0.0 (0.0)
Other	0 (0.0)	1 (3.2)
BMI, mean (SD), kg/m^2^	31.4 (9.5) (missing: 6)	24.4 (4.0) (missing: 1)
Smoking status, *n* (%)		
None/former	27 (87.1)	31 (100.0)
Active	1 (3.2) (missing: 3)	0 (0.0)
Psychological variables		
PHQ-9 scores, mean (SD)	12.8 (5.3) (missing: 6)	2.8 (2.8)

**Table 2:** Summary of clinical characteristics and symptom profile of the Long COVID (LC) patient group.

Clinical characteristics	
Duration of LC symptoms, mean (SD)	16.1 months (7.6)
Number of COVID-19 infections, *n* (%)	
infected once	13 (43.3)
infected twice	14 (46.7)
infected 3 times	3 (10) (missing: 1)
Pre-existing comorbidities (prior LC), *n* (%)	
type-1 or type-2 diabetes mellitus	0 (0)
hypertension	0 (0)
chronic respiratory conditions	3 (12.5)
chronic heart disease	1 (4.2)
chronic neurological conditions	1 (4.2)
chronic musculoskeletal conditions	2 (8.3)
chronic kidney disease	0 (0)
chronic liver disease	0 (0)
no pre-existing medical conditions	7 (29.2)
other	15 (62.5) (missing: 7)
Medication, *n* (%)	
Paracetamol	21 (70)
NSAIDs	14 (46.7)
Opioids	4 (13.3)
Gabapentinoids	2 (6.7)
SSRIs	3 (10)
SNRIs	0 (0)
Tricyclic antidepressants	2 (6.7)
Antihistamines	1 (3.3)
Beta-blockers	2 (6.7)
H_2_ receptor antagonists	2 (6.7)
Proton-pump inhibitors	2 (6.7)
no medication	4 (13.3) (missing: 7)
Long COVID symptoms	
C19-YRS items related to neurological and psychological symptoms of LC (missing: 4)	Number of participants reporting symptom (%), mean post-COVID severity (SD)
Fatigue	27 participants (100%), 8.2 (1.4)
Pain	27 participants (100%), 6.8 (1.4)
Cognition	25 participants (92.6%), 6.4 (2.3)
Anxiety	25 participants (92.6%), 6.1 (2.5)
Depression	19 participants (70.4%), 6.0 (2.1)
Overall symptom severity, mean (SD)	45.1 (17.4) (missing: 4)

### Association between PAF and pain severity in LC patients with new-onset chronic pain

A negative association was observed between global PAF (averaged across all electrode sites) and pain severity scores, but did not withstand adjustment for confounders (standardised *β* = −0.336, 95% CI [−0.78, 0.11], F(3,21) = 1.85, adj. *R*^2^ = 0.096, FDR-corrected *p* = 0.222 for max-PAF; standardised *β* = −0.327, 95% CI [−0.75, 0.10], F(3,21) = 1.88, adj. *R*^2^ = 0.099, FDR-corrected *p* = 0.229 for aperiodic-adjusted PAF) (Supplementary Table S2).

**Figure 1:**
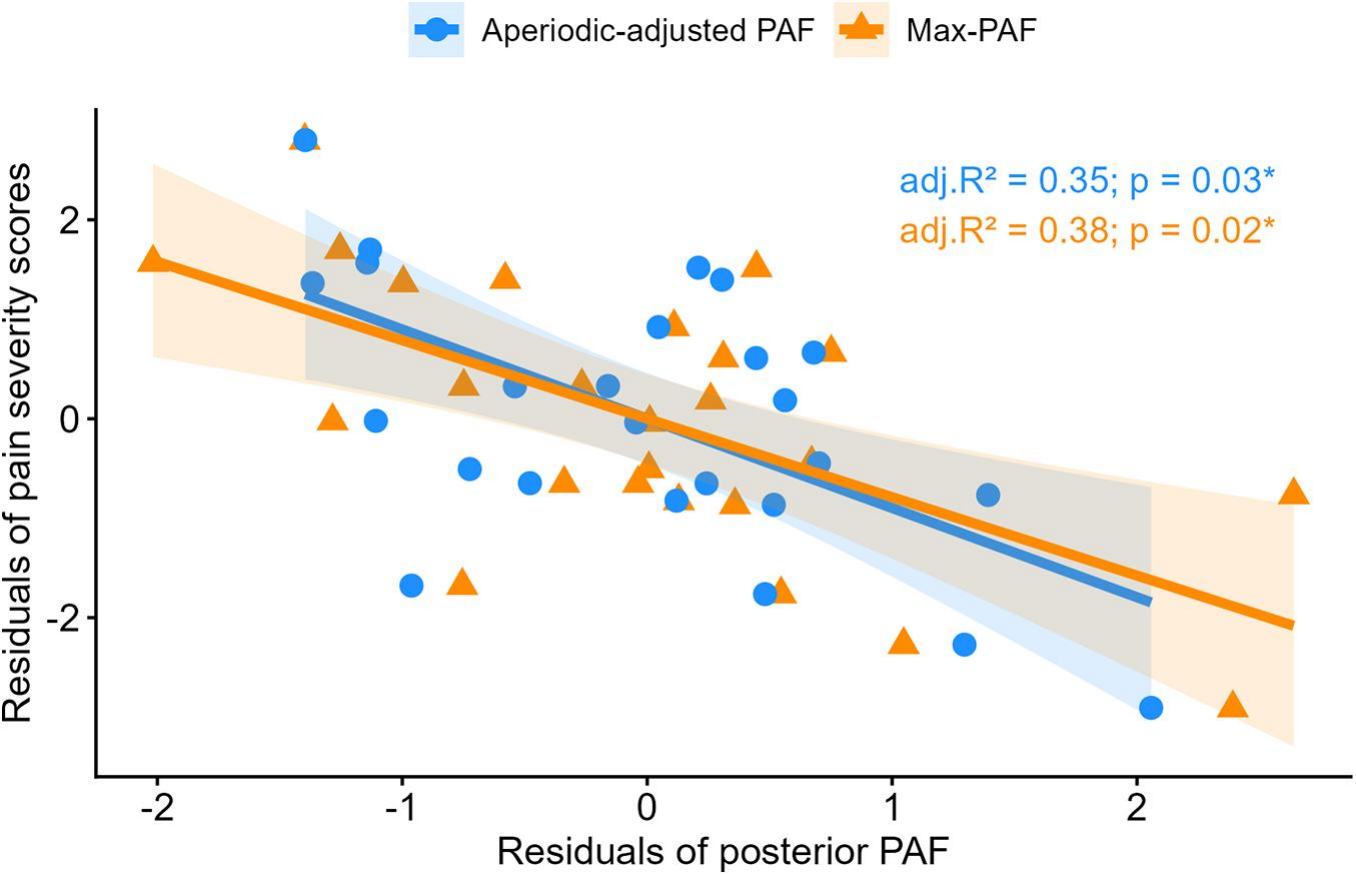
Partial regression plot showing association between peak alpha frequency (PAF) over posterior scalp region and pain severity in Long COVID patients with new-onset chronic pain, controlling for the effects of age and depression. Each point represents a residual of C19-YRS pain severity scores (subscale of C19-YRS) after removing covariate effects, plotted against the residual of posterior PAF after removing the effects of the same covariates. Both max-PAF and aperiodic-adjusted PAF estimates are included in the plot.

Regional analysis indicated a significant relationship between pain severity and PAF over posterior electrode sites, even when adjusting for the potential confounding effects of age and depression (Table 3). The effect of introducing each confounding variable to the regression models is detailed in Supplementary Table S2. More specifically, lower posterior PAF estimates were associated with greater pain severity scores on the C19-YRS [standardised *β* = −0.602, 95% CI [−0.95, −0.26], coefficient *p* = 0.001 for max-PAF; standardised *β* = −0.581, 95% CI [−0.93, −0.23], coefficient *p* = 0.002 for aperiodic-adjusted PAF] (Figure 1). This finding was consistent across methods of estimation of PAF [F(3,21) = 5.94, adj. *R*^2^ = 0.382, FDR-corrected *p* = 0.017 for max-PAF; F(3,21) = 5.35, adj. *R*^2^ = 0.352, FDR-corrected *p* = 0.027 for aperiodic-adjusted PAF]. The independent metrics (PAF, age, depression) were not correlated between each other and in combination accounted for approximately 40% variance in pain intensity. No such association was observed for anterior (standardised *β* = −0.292, 95% CI [−0.74, 0.16], F(3,21) = 1.59, adj. *R*^2^ = 0.069, FDR-corrected *p* = 0.222 for max-PAF; standardised *β* = −0.163, 95% CI [−0.59, 0.27], F(3,21) = 1.15, adj. *R*^2^ = 0.018, FDR-corrected *p* = 0.352 for aperiodic-adjusted PAF) and central scalp regions (standardised *β* = −0.340, 95% CI [−0.78, 0.10], F(3,21) = 1.87, adj. *R*^2^ = 0.098, FDR-corrected *p* = 0.222 for max-PAF; standardised *β* = −0.326, 95% CI [−0.76, 0.11], F(3,21) = 1.83, adj. *R*^2^ = 0.094, FDR-corrected *p* = 0.229 for aperiodic-adjusted PAF).

**Table 3:** Summary of multiple linear regression models on the association between posterior peak alpha frequency (PAF) and pain severity, controlling for the effects of age and depression.

Model outputs	Max-PAF	Aperiodic-adjusted PAF
Posterior PAF	**−0**.602**	**−0**.581**
	(0.165)	(0.169)
Age	−0.035	0.059
	(0.161)	(0.169)
Depression	0.199	0.268
	(0.165)	(0.167)
Constant	<0.001***	<0.001***
	(0.157)	(0.161)
*R* ^2^	0.459	0.433
Adj. *R*^2^	0.382	0.352
Residual SE	0.786	0.805
F Statistic	5.94 (df = 3;21)	5.35 (df = 3;21)
Corrected *p*	**0** **.** **017** *	**0** **.** **027** *

Models derived from max-PAF and aperiodic-adjusted PAF estimates are shown. Standardised *β* coefficients and standard errors for the independent variable and confounders, *R*^2^, adjusted *R*^2^, residual standard error (SE), F statistic and degrees of freedom, and false discovery rate (FDR)-corrected *p* values are reported for each model.

* *p* < 0.05.

** *p* < 0.01.

*** *p* < 0.001.Boldface indicates regression coefficients (*β*) corresponding to independent variables with a statistically significant independent association with the outcome, as well as models with statistically significant overall *p* values.

A sensitivity analysis accounting for the potential confounding effects of use of centrally acting medication showed the observed association between posterior PAF and pain severity was not accounted for by medication status (standardised *β* for PAF = −0.618, 95% CI [−1.09 −0.14], F(4,20) = 4.30, adj. *R*^2^ = 0.355, *p* = 0.011 for max-PAF; standardised *β* = −0.593, 95% CI [−1.17 −0.02], F(4,20) = 3.85, adj. *R*^2^ = 0.322, *p* = 0.018 for aperiodic-adjusted PAF) (Table 4).

**Table 4:** Summary of sensitivity analysis models accounting for the potential confounding effects of use of centrally acting medication on the association between posterior PAF and pain severity.

Model outputs	Max-PAF	Aperiodic-adjusted PAF
Posterior PAF	**−0** **.** **618** **	**−0** **.** **593** **
	(0.174)	(0.179)
Age	−0.032	0.063
	(0.165)	(0.173)
Depression	0.209	0.277
	(0.171)	(0.173)
Centrally acting drugs	−0.060	−0.046
	(0.174)	(0.177)
Constant	<0.001***	<0.001***
	(0.161)	(0.165)
*R* ^2^	0.462	0.435
Adj. *R*^2^	0.355	0.322
Residual SE	1.158	1.186
F Statistic	4.30 (df = 4;20)	3.85 (df = 4;20)
Model *p* value	**0** **.** **011** *	**0** **.** **018** *

Medication status was added as binary factor (taking centrally acting medication = 1, not taking centrally acting medication = 0) to the models. Models derived from max-PAF and aperiodic-adjusted PAF estimates are shown. Standardised *β* coefficients and standard errors for the independent variable and confounders, *R*^2^, adjusted *R*^2^, residual standard error, F statistic and degrees of freedom, and model *p* values are reported for each model.

* *p* < 0.05.

** *p* < 0.01.

*** *p* < 0.001.Boldface indicates regression coefficients (*β*) corresponding to independent variables with a statistically significant independent association with the outcome, as well as models with statistically significant overall *p* values.

**Figure 2:**
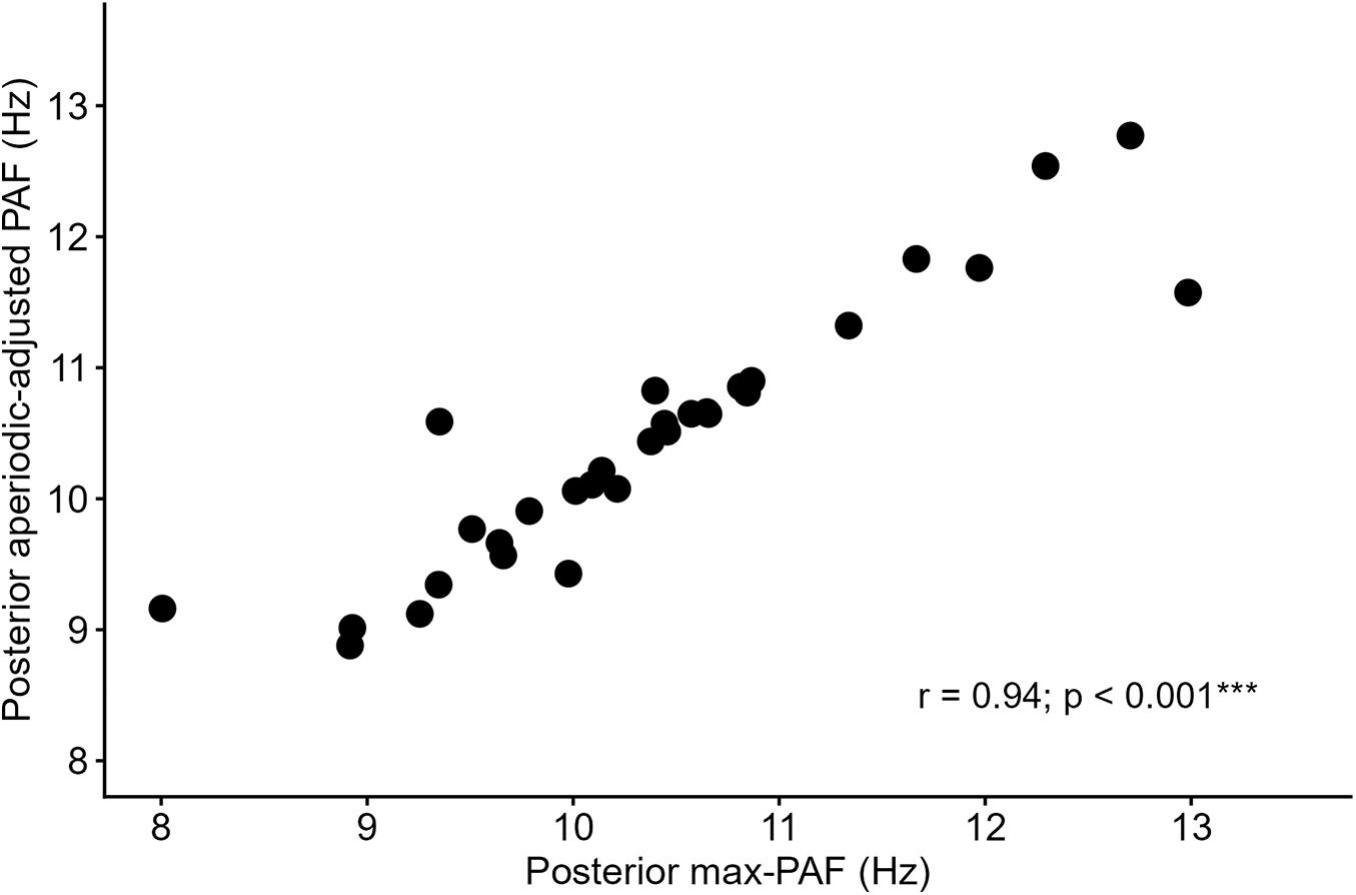
Scatterplot showing strong correlation between peak alpha frequency (PAF) estimates (posterior region) derived from two methods: frequency in the alpha range with maximum Welch's spectral power (max-PAF) and frequency with the strongest Gaussian peak identified in the alpha range with spectral parameterisation algorithm (aperiodic-adjusted PAF). Correlation coefficient rho and *p* value shown are based on Spearman's rank correlation.

Due to consistency of findings across methods of PAF extraction and strong correlation between max-PAF and aperiodic-adjusted PAF estimates (*r* = 0.943, *p* < 0.001 for posterior PAF; Figure 2) (see Supplementary Table S3 for other regions), subsequent analyses results are reported here for aperiodic-adjusted PAF estimates only, unless otherwise stated. Summary tables detailing results from both methods can be found in Supplementary materials.

We also tested whether PAF relates to other neurological symptoms frequently reported in LC and other pain-related metrics. Table 5 provides a summary of model outputs, with full model results presented in Supplementary Table S4. No evidence was found for a relationship between posterior PAF and severity of fatigue [standardised *β* = −0.284, 95% CI [−0.70, 0.13], F(3,21) = 1.85, adj. *R*^2^ = 0.096, *p* = 0.168] or pain widespreadness [standardised *β* = −0.038, 95% [−0.51, 0.44], F(3,21) = 0.10, adj. *R*^2^ = −0.132, *p* = 0.957]. Furthermore, the results show that posterior PAF did not independently predict severity of cognitive impairments and overall LC symptom severity, with any models that reached significance threshold resulting from the confounding effects of depression (standardised *β* for PAF = −0.116, 95% CI [−0.50, 0.26], F(3,21) = 3.60, adj. *R*^2^ = 0.245, *p* = 0.030 for cognitive impairments; standardised *β* for PAF = −0.216, 95% CI [−0.50, 0.07], F(3,21) = 11.91, adj. *R*^2^ = 0.577, *p* < 0.001 for overall symptom severity) (Table 5). The data indicated a possible negative association between posterior PAF and duration of pain [standardised *β* = −0.447, 95% CI [−0.83, −0.06], F(3,21) = 3.44, adj. *R*^2^ = 0.234, *p* = 0.035 for aperiodic-adjusted PAF], however it was not sustained for max-PAF estimates after adjusting for confounders [standardised *β* = −0.361, 95% CI [−0.76, 0.04], F(3,21) = 2.53, adj. *R*^2^ = 0.160, *p* = 0.085 for max-PAF].

**Table 5:** Summary of multiple linear regression models on the association between posterior peak alpha frequency (PAF), other self-reported neurological symptoms of LC (fatigue and cognitive symptoms) and other pain-related metrics (pain duration and pain widespreadness).

Model outputs	Severity of other neurological symptoms		Other pain-related metrics		Overall symptom severity
	Fatigue	Cognition	Duration	Widespreadness	
Posterior PAF	−0.284	−0.116	−0.447*	−0.038	−0.216
	(0.200)	(0.183)	(0.184)	(0.228)	(0.137)
Age	−0.201	0.043	0.333	−0.024	−0.090
	(0.199)	(0.182)	(0.183)	(0.229)	(0.136)
Depression	0.233	**0** **.** **557** **	0.231	−0.113	**0** **.** **735** ***
	(0.196)	(0.182)	(0.180)	(0.226)	(0.134)
Constant	<0.001***	<0.001***	<0.001***	<0.001***	<0.001***
	(0.190)	(0.174)	(0.175)	(0.217)	(0.130)
*R* ^2^	0.209	0.339	0.330	0.015	0.630
Adj. *R*^2^	0.096	0.245	0.234	−0.132	0.577
Residual Std. Error	0.950	0.339	0.875	1.064	0.650
F Statistic	1.85 (df = 3;21)	3.60 (df = 3;21)	3.44 (df = 3;21)	0.10 (df = 3;20)	11.91 (df = 3;21)
Model *p* value	0.168	**0** **.** **030** *	**0** **.** **035** *	0.957	**<0** **.** **001** ***

The models represented in the table are derived from aperiodic-adjusted PAF estimates. Standardised *β* coefficients and standard errors for the independent variable and confounders, *R*^2^, adjusted *R*^2^, residual standard error (SE), F statistic and degrees of freedom, and false discovery rate (FDR)-corrected *p* values are reported for each model.

* *p* < 0.05.

** *p* < 0.01.

*** *p* < 0.001.Boldface indicates regression coefficients (*β*) corresponding to independent variables with a statistically significant independent association with the outcome, as well as models with statistically significant overall *p* values.

### Association between alpha band power and pain severity in LC patients with new-onset chronic pain

The results show no association between alpha band power and severity of pain symptoms at the global or regional level (Table 6). Detailed model outputs can be found in Supplementary Table S5.

**Table 6:** Summary of linear regression models on the association between alpha band power and pain severity.

Model outputs	Global	Anterior	Central	Posterior
Alpha power spectral density	−0.007	0.043	−0.060	−0.065
	(0.201)	(0.217)	(0.221)	(0.214)
Age	−0.073	−0.058	−0.093	−0.089
	(0.219)	(0.216)	(0.220)	(0.214)
Depression	0.337	0.331	0.344	0.342
	(0.208)	(0.207)	(0.207)	(0.206)
Constant	<0.001	<0.001	<0.001	<0.001
	(0.201)	(0.201)	(0.201)	(0.201)
*R*²	0.116	0.117	0.119	0.119
Adj. *R*²	−0.011	−0.009	−0.007	−0.006
Residual Std. Error	1.005	1.004	1.004	1.003
F Statistic	0.92 (df = 3;21)	0.93 (df = 3;21)	0.94 (df = 3;21)	0.95 (df = 3;21)
FDR-corrected *p* value	0.450	0.450	0.450	0.450

Models derived from global and regional (including anterior, central and posterior regions) alpha band power estimates are shown. Standardised *β* coefficient and standard error for the independent variable and confounders, *R*^2^, adjusted *R*^2^, residual standard error, F statistic and degrees of freedom, and false discovery rate (FDR)-corrected *p* values are reported for each model.

As exploratory analysis, power spectral density of the strongest peak within the alpha range (PAF power spectral density) was also tested as a predictor, but it did not show a significant association with pain severity (standardised *β* = 0.050, 95% CI [−0.39, 0.49], F(3,21) = 0.94, adj. *R*^2^ = −0.008, *p* = 0.441 for max-PAF; standardised *β* = 0.009, 95% CI [−0.44, 0.46], F(3,21) = 0.92, adj. *R*^2^ = −0.011, *p* = 0.450 for aperiodic-adjusted PAF).

### Comparative analysis of PAF between LC patients with new-onset chronic pain and healthy pain-free controls

We next examined whether LC patients exhibited lower PAF compared to healthy pain-free controls across all predefined regions using multiple linear regression models, with a grouping factor and age as confounding variable. No evidence of significant differences was found between the LC patient group and the healthy pain-free control group for global PAF [F(1,59) = 2.60, *p* = 0.112] or PAF from any of the regions tested (*p* ≥ 0.054) (see Supplementary Table S7 for detailed 2-group model outputs).

### Comparative analysis of alpha band power between LC patients with new-onset chronic pain and healthy pain-free controls

Alpha band power did not differ between the LC patient group and the control group at any of the regions tested (*p* ≥ 0.760) (see Supplementary Table S9 for detailed 2-group model outputs).

## Discussion

To our knowledge, this is the first study to investigate EEG alpha oscillatory features associated with pain symptomology in a cohort of LC patients with new-onset chronic pain. Consistent with our hypothesis, we found that lower PAF is associated with higher severity of LC-chronic pain. This result was localised to the posterior scalp region and consistent across different PAF estimation methods. No differences in PAF were found between LC patient group and healthy pain-free control group. Our results on alpha band power show no association with pain severity, as well as no differences between groups.

### Association between new-onset LC-chronic pain and EEG alpha oscillatory features

While studies have shown decreased PAF in chronic pain patients, evidence of an inverse relation between PAF and pain severity comes mostly from human experimental pain studies (31, 32, 56). Our findings extend the literature on the PAF-pain association to a novel post-infection pain syndrome. Here we report that lower PAF in the posterior scalp region is associated with higher severity of LC-chronic pain, independent of age, depression and the effects of centrally acting medication on PAF. Alpha oscillations are thought to play a role in the dynamic gating and processing of sensory information by functionally inhibiting task- or stimulus-irrelevant cortical regions (57). Thus, slower alpha activity may reflect impaired ability to modulate incoming pain information contributing to heightened pain severity (31, 32).

The regional localisation of our finding could be explained by alpha rhythms being most prominent over occipital-parietal scalp region, supporting the idea that posterior scalp sites are where alpha/PAF differences and behaviour associations are most evident (58). This is corroborated by previous research showing differences in PAF between chronic pain patients and pain-free controls are more pronounced in the occipital-parietal areas (25, 59). Additionally, the observed regional differences may reflect distinct but overlapping neurophysiological generators of alpha rhythms. Despite sharing a similar frequency range, the posterior dominant alpha rhythm is maximal over the occipital cortex and primarily associated with visual processing, whereas mu rhythm is more centred over the sensorimotor cortex and modulated by movement and somatosensory input (60). The scalp-level posterior effects could reflect contributions from multiple alpha-generating networks and should not be interpreted as a pain-specific neural generator.

Max-PAF and aperiodic-adjusted PAF estimates were highly correlated, as previously shown by Deodato et al. (61) and Lebrun et al. (62), and yielded comparable results. This supports the robustness of our findings across methods and indicates that the observed association is not driven by aperiodic 1/f-like activity. Notably, Lebrun et al. (62) and Fathi et al. (63), both in the context of PAF-pain association research, further addressed this issue using power spectrum subtraction prior to PAF estimation, likewise supporting the stability of PAF metrics. Together, these findings strengthen the conclusion that aperiodic activity does not account for the observed association.

Furthermore, we show that posterior PAF is not associated with severity of fatigue, cognitive impairments, and overall LC symptom severity, providing evidence to support the symptom-specificity of our findings. This builds upon the framework outlined in our prior review of quantitative EEG studies on LC and similar multisymptom clinical syndromes (40), where we propose a symptom-focused approach to LC research. The observed negative association between pain duration and posterior PAF aligns with the observations by de Vries et al. (25). Nevertheless, this exploratory finding is unlikely to withstand correction for multiple testing and needs confirming in future longitudinal studies.

### Comparison between LC patients with new-onset chronic pain and pain-free controls

To date, no study has investigated the association between LC pain and EEG oscillatory activity. Limited prior work has investigated alpha oscillatory cortical neural activity in LC (without emphasis on pain). These studies focused mainly on post-COVID cognitive disturbances and reported mixed results of reduced (64), increased (65) or no difference (66, 67) in alpha power/current source density in patients compared to controls or EEG recordings from the same individuals recorded prior to developing LC. Only a single study has incorporated PAF in the analysis, with findings suggesting reduced occipital PAF in patients with post-COVID cognitive disturbances compared to controls (66).

Our study found no differences in PAF or alpha band power between the overall LC patient group and controls. Several plausible factors could account for the observed lack of differences: (a) there are effectively no differences in EEG alpha oscillatory features between groups; (b) there are subtle differences between groups, although the small sample size limits the ability to detect them; and (c) heterogeneity within the patient group could mask potential neurophysiological differences that are present only in a subset of patients.

One possible explanation for the observed PAF-pain association in LC patients but lack of differences relative to controls is that PAF relates to interindividual differences in pain severity within LC, rather than representing a neurophysiological abnormality specific to LC-associated chronic pain. In support of this possibility, exploratory subgroup analysis using a median split (Supplementary materials) suggested potential differences in PAF between pain severity-based subgroups. More specifically, LC patients with moderate pain tended to show heightened PAF on average compared to pain-free controls, while those with severe pain showed reduced posterior PAF relative to moderate pain subgroup, but not relative to controls. Nevertheless, given the exploratory nature of these analyses, these observations should be interpreted with caution and regarded as hypothesis-generating, pending replication in adequately powered and prospectively designed studies. Moreover, due to the cross-sectional design, we cannot determine the temporal or causal nature of this association. For example, it is unclear whether slower PAF reflects a pre-existing susceptibility to develop more severe pain upon contraction of COVID-19 infection or a consequence of ongoing pain. Prior studies suggest that resting-state pain-free PAF may be associated with severity of prolonged pain in experimental tonic pain models and clinical post-operative pain (31–34). Testing whether this applies in LC would require longitudinal data, ideally including pre-infection baseline EEG which are not available for this cohort (31–34).

An alternative, also untested possibility is that the observed patterns across patients may reflect state-dependent neurophysiological changes associated with fluctuations in pain over time. Though plausible in view of the evidence on PAF-pain association, studies have failed to demonstrate that PAF tracks changes in pain over time (32, 68) reinforcing prior reports of PAF as a relatively stable neurophysiological trait marker (28, 69). These explanations highlight the need for longitudinal studies to clarify the relationship between PAF and pain dynamics in LC.

Finally, clinical heterogeneity among LC patients may also contribute to reduced sensitivity to detect group-level differences. In our cohort, we have previously reported that 72.2% of patients met diagnostic criteria for FMS and 85% showed signs of central sensitisation based on quantitative sensory testing, supporting the presence of nociplastic type pain (14, 18); however, this was not universal. It is possible that the remaining proportion of patients present with pain associated with different mechanistic features, with likely dominance of nociceptive and neuropathic contributors which can be associated with different neurophysiological patterns. For example, studies on chronic pain syndromes of neuropathic origin have reported increases in alpha power (26, 38, 39), in contrast to observations of reduced alpha in FMS (35–37). Such heterogeneity may introduce variability and obscure group-level differences, particularly in a relatively state-dependent variable EEG measure such as alpha power. Further to this, heterogeneity could also be attributed, at least in part, to the presence of distinct LC symptom phenotypes. This possibility merits further investigation in phenotype-specific cohorts, either in separate studies or combined for comparative analysis.

### Limitations

This study has several limitations that should be considered when interpreting the findings. First, the small sample size may limit generalization of findings. We specifically recruited LC patients without pre-existing musculoskeletal chronic pain prior to COVID-19 infection in an effort to prevent confounding effects of pain of different aetiology, which introduced an additional challenge to recruitment process and limited the pool of potential participants. Second, eyes-open resting-state EEG was selected to reduce drowsiness and maintain attention, which is particularly important in the LC population where fatigue and sleep disturbances may influence alertness. While this approach is methodologically valid, it should be noted that alpha power is typically higher in the eyes-closed condition. In contrast, although small shifts in PAF may occur in some individuals, prior work suggests that PAF is generally considered more stable across eyes-open and eyes-closed recordings (29, 70). Nonetheless, the use of an eyes-open paradigm may limit direct comparability with prior studies. Future studies should also incorporate objective measures of state drowsiness to better account for fluctuations in alertness during EEG recording and their potential influence on alpha features. Third, we acknowledge the potential for confounding by other factors not measured or modelled in this study, such as sleep, anxiety and comorbidities. While the groups were matched for age and sex, we note that other demographic and clinical differences between patients and controls, including potentially higher BMI in patients, may have contributed residual confounding in the comparative analyses. Finally, in our exploratory supplementary analysis, subgrouping patients based on pain severity may reduce statistical power and introduce classification bias. On this note, our data-driven median cut-off of 7 between moderate and severe pain subgroups closely aligns with empirically derived thresholds suggested in chronic pain literature (71–74) and often used in clinic. Nevertheless, the results from subgroup analysis should be regarded as exploratory and preliminary, warranting further validation in larger cohorts.

### Conclusions and recommendations for future research

The findings of this study provide evidence to support posterior PAF as an objective, measurable neurophysiological brain feature that associates with severity of new-onset LC-chronic pain. This work contributes to the growing body of evidence suggesting, among other factors, a neurophysiological basis of chronic pain symptoms in LC, a condition that to date remains the subject of stigma and associations with psychosomatisation. Future research should incorporate a multidimensional assessment of pain, capturing affective, cognitive and behavioural domains, to provide a more comprehensive understanding of the relationship between neurophysiological correlates and the broader experience of post-COVID pain. While preliminary, our observations of subgroup differences in PAF may inform the design of larger, longitudinal studies to assess how PAF dynamically evolves alongside the natural progression of pain and to further elucidate variability within the LC patient group. Overall, our findings, while early, suggest non-invasive targeted neuromodulation could be explored as a potential therapeutic avenue for new-onset LC chronic pain. Tailored neuromodulation strategies show promise within an integrated multidisciplinary management framework for LC-chronic pain, and could be relevant to other similar post-infectious syndromes manifesting with chronic pain symptomology.

## Data Availability

The raw data supporting the conclusions of this article will be made available by the authors, without undue reservation.
