# Supplementary material for: Peak alpha frequency is associated with pain severity in Long COVID patients with new-onset chronic pain
Source: Front Pain Res (Lausanne). 2026 Jul 29;7:1893610. doi: 10.3389/fpain.2026.1893610 (PMC13461798; doi:10.3389/fpain.2026.1893610)
Supplement: Supplementary file 1 [file Datasheet1.pdf]

Supplementary materials

Manuscript title: Peak alpha frequency is associated with pain severity in Long COVID patients with new-onset chronic pain

Authors: Bárbara Silva-Passadouro[1], Omar Khoja[1,2], Alexander J. Casson[3], Ioannis Delis[4,5], Christopher Brown[6], Manoj Sivan[1,7*]

1 Leeds Institute of Rheumatic and Musculoskeletal Medicine, School of Medicine, University of Leeds, Leeds, UK

2 King Salman Bin Abdulaziz Medical City, Al-Madinah, Saudi Arabia

3 Department of Electrical and Electronic Engineering, University of Manchester, Manchester, UK

4 School of Biomedical Sciences, Faculty of Biological Sciences, University of Leeds, Leeds, UK

5 School of Electrical and Computer Engineering, National Technical University of Athens, Athens, Greece

6 Department of Psychology, Institute of Population Health, University of Liverpool, Liverpool, UK

7 National Demonstration Centre in Rehabilitation Medicine, Leeds Teaching Hospitals NHS Trust, Leeds, UK

*Correspondence:

Manoj Sivan, Leeds Institute of Rheumatic and Musculoskeletal Medicine, 2nd Floor, Chapel Allerton Hospital, Chapeltown Road, Leeds, LS7 4SA

Email: M.Sivan@leeds.ac.uk

Table S1. Summary of pre- and post-COVID symptom severity in the Long COVID patient group based C19-YRS items.

**Long COVID symptoms**		
C19-YRS items (missing: 4)	Pre-COVID	Post-COVID
Breathlessness - at rest	4 participants (14.8%)	17 participants (63.0%)
mean severity (SD)	4.2 (3.3)	4.6 (2.8)
Breathlessness - dressing	6 participants (22.2%)	23 participants (85.2%)
mean severity (SD)	2.8 (1.8)	4.9 (2.9)
Breathlessness - stairs	13 participants (48.1%)	26 participants (96.3%)
mean severity (SD)	1.9 (0.9)	6.2 (2.6)
Cough/throat sensitivity/voice change	5 participants (18.5%)	19 participants (70.4%)
mean severity (SD)	3.2 (2.6)	5.4 (2.6)
Fatigue	16 participants (59.3%)	27 participants (100%)
mean severity (SD)	2.1 (0.8)	8.2 (1.4)
Continence	5 participants (18.5%)	13 participants (48.1%)
mean severity (SD)	2.0 (1.7)	5.6 (2.2)
Pain	0 participants (0%)	27 participants (100%)
mean severity (SD)	0.0 (0.0)	6.8 (1.4)
Cognition	12 participants (44.4%)	25 participants (92.6%)
mean severity (SD)	2.5 (1.4)	6.4 (2.3)
Anxiety	16 participants (59.3%)	25 participants (92.6%)
mean severity (SD)	2.7 (1.6)	6.1 (2.5)
Depression	9 participants (33.3%)	19 participants (70.4%)
mean severity (SD)	3.4 (2.3)	6.0 (2.1)
PTSD	5 participants (18.5%)	16 participants (59.3%)
mean severity (SD)	1.8 (1.8)	5.4 (2.5)
Communication	6 participants (22.2%)	20 participants (74.1%)
mean severity (SD)	1.7 (0.8)	5.6 (2.6)
Mobility	5 participants (18.5%)	21 participants (77.8%)
mean severity (SD)	1.8 (1.8)	5.9 (2.3)
Personal care	2 participants (7.4%)	14 participants (51.8%)
mean severity (SD)	3.5 (3.5)	3.9 (2.4)
Other activities of daily living	5 participants (18.5%)	25 participants (92.6%)
mean severity (SD)	1.6 (0.9)	7.1 (1.8)
Social role	2 participants (7.4%)	25 participants (92.6%)
mean severity (SD)	1.0 (0.0)	5.9 (2.3)
C19-YRS symptom severity, mean (SD)	7.2 (8.2)	45.1 (17.4)
C19-YRS functional disability, mean (SD)	1.3 (2.9)	22.8 (10.9)
C19-YRS global health score, mean (SD)	8.0 (1.2)	2.7 (1.6)

Table S2. Summary of multiple linear regression models on the association between peak alpha frequency (PAF) and pain severity. Model 1 represents results of simple linear regression between dependent variable (pain severity) and independent variable (PAF). Models 2 and 3 outline the effects of introducing each confounding variable (age and depression, respectively); and Model 4 includes confounding effects of both age and depression. Models derived from max-PAF and aperiodic-adjusted PAF estimates are shown. Standardised β coefficients and 95% confidence intervals (CI) for each independent variable, F statistic and degrees of freedom, adjusted R^2, raw overall model p values and false discovery rate (FDR)-corrected p values are reported for each combination of PAF extraction method (max-PAF and aperiodic-adjusted PAF), scalp region (global, anterior, central and posterior region), and model (model 1 to model 4). Only the p values from the models adjusted for both confounders (model 4) were adjusted for multiple testing using FDR correction. * $p < 0.05$; ** $p < 0.01$; *** $p < 0.001$.

	**Standardised β coefficient for PAF [95% CI]**	**Standardised β coefficient for age [95% CI]**	**Standardised β coefficient for depression [95% CI]**	**F statistic (degrees of freedom)**	**Adj. R^2**	**Overall model p value**	**FDR-corrected p value**
**Global**							
**max-PAF models**							
Model 1	**-0.402*** [-0.80 -0.01]			4.42 (1,23)	0.125	**0.047***	---
Model 2	**-0.417*** [-0.82 -0.01]	-0.111 [-0.52 0.29]		2.31 (2,22)	0.098	0.123	---
Model 3	-0.320 [-0.75 0.11]		0.206 [-0.22 0.64]	2.70 (2,22)	0.124	0.089	---
Model 4	-0.336 [-0.78 0.11]	-0.111 [-0.52 0.30]	0.206 [-0.23 0.64]	1.85 (3,21)	0.096	0.168	0.222
**aperiodic-adjusted PAF models**							
Model 1	**-0.401*** [-0.80 -0.01]			4.42 (1,23)	0.125	**0.047***	---
Model 2	-0.401 [-0.81 0.01]	-0.050 [-0.45 0.35]		2.15 (2,22)	0.088	0.140	---
Model 3	-0.329 [-0.74 0.09]		0.228 [-0.19 0.64]	2.88 (2,22)	0.136	0.077	---
Model 4	-0.327 [-0.75 0.10]	-0.063 [-0.47 0.34]	0.231 [-0.19 0.66]	1.88 (3,21)	0.099	0.164	0.229
**Anterior region**							
**max-PAF models**							
Model 1	-0.375 [-0.77 0.02]			3.77 (1,23)	0.103	0.064	---
Model 2	-0.382 [-0.79 0.03]	-0.084 [-0.49 0.33]		1.91 (2,22)	0.070	0.172	---
Model 3	-0.286 [-0.73 0.16]		0.211 [-0.23 0.65]	2.37 (2,22)	0.103	0.117	---
Model 4	-0.292 [-0.74 0.16]	-0.088 [-0.50 0.32]	0.213 [-0.24 0.67]	1.59 (3,21)	0.069	0.222	0.222
**aperiodic-adjusted PAF models**							
Model 1	-0.231 [-0.65 0.19]			1.29 (1,23)	0.012	0.267	---
Model 2	-0.228 [-0.66 0.20]	-0.037 [-0.47 0.39]		0.63 (2,22)	-0.031	0.539	---
Model 3	-0.168 [-0.59 0.25]		0.297 [-0.12 0.72]	1.76 (2,22)	0.059	0.196	---
Model 4	-0.163 [-0.59 0.27]	-0.057 [-0.48 0.37]	0.301 [-0.13 0.73]	1.15 (3,21)	0.018	0.352	0.352
**Central region**							
**max-PAF models**							
Model 1	**-0.408*** [-0.80 -0.01]			4.60 (1,23)	0.131	**0.043***	---
Model 2	**-0.421*** [-0.82 -0.02]	-0.104 [-0.51 0.30]		2.37 (2,22)	0.103	0.116	---
Model 3	-0.327 [-0.76 0.11]		0.199 [-0.23 0.63]	2.75 (2,22)	0.127	0.086	---

Model 4	-0.340 [-0.78 0.10]	-0.105 [-0.51 0.30]	0.200 [-0.24 0.64]	1.87 (3,21)	0.098	0.165	0.222
**aperiodic-adjusted PAF models**							
Model 1	**-0.404*** [-0.80 -0.01]			4.50 (1,23)	0.127	**0.045***	---
Model 2	**-0.405*** [-0.81 -0.01]	-0.058 [-0.46 0.34]		2.20 (2,22)	0.091	0.134	---
Model 3	-0.326 [-0.75 0.10]		0.213 [-0.21 0.64]	2.80 (2,22)	0.131	0.082	---
Model 4	-0.326 [-0.76 0.11]	-0.069 [-0.47 0.34]	0.217 [-0.22 0.65]	1.83 (3,21)	0.094	0.172	0.229
**Posterior region**							
**max-PAF models**							
Model 1	**-0.649***** [-0.98 -0.32]			16.72 (1,23)	0.396	**<0.001*****	---
Model 2	**-0.648***** [-0.98 -0.31]	-0.022 [-0.36 0.31]		8.01 (2,22)	0.369	**0.002****	---
Model 3	**-0.605**** [-0.94 -0.27]		0.197 [-0.14 0.53]	9.28 (2,22)	0.408	**0.001****	---
Model 4	**-0.602**** [-0.95 -0.26]	-0.035 [-0.37 0.30]	0.199 [-0.14 0.54]	5.94 (3,21)	0.382	**0.004****	**0.017***
**aperiodic-adjusted PAF models**							
Model 1	**-0.597**** [-0.94 -0.25]			12.74 (1,23)	0.328	**0.002****	---
Model 2	**-0.614**** [-0.98 -0.25]	0.080 [-0.28 0.44]		6.26 (2,22)	0.305	**0.007****	---
Model 3	**-0.568**** [-0.90 -0.23]		0.273 [-0.06 0.61]	8.30 (2,22)	0.378	**0.002****	---
Model 4	**-0.581**** [-0.93 -0.23]	0.059 [-0.29 0.41]	0.268 [-0.08 0.61]	5.35 (3,21)	0.352	**0.007****	**0.027***

Table S3. Correlation between max-PAF estimates and aperiodic-adjusted PAF estimates derived from global, anterior, central and posterior regions. *** $p < 0.001$.

Region	Spearman's rho	p value
Global	0.960	**<0.001*** **
Anterior	0.925	**<0.001*** **
Central	0.975	**<0.001*** **
Posterior	0.943	**<0.001*** **

Table S4. Summary of multiple linear regression models on the association between posterior peak alpha frequency (PAF), other self-reported neurological symptoms of Long COVID, pain-related metrics and overall symptom severity. Model 1 represents results of simple linear regression between dependent variable (severity of fatigue, severity of cognitive symptoms, pain duration, pain widespreadness, overall LC symptom severity) and independent variable (PAF over posterior scalp region). Models 2 and 3 outline the effects of introducing each confounding variable (age and depression, respectively); and Model 4 includes confounding effects of both age and depression. Models derived from max-PAF and aperiodic-adjusted PAF estimates are shown. Standardised β coefficients and 95% confidence intervals (CI) for each independent variable, F statistic and degrees of freedom, adjusted R^2 and raw overall model p values are reported for each combination of dependent variable tested (severity of fatigue, severity of cognitive symptoms, pain duration, pain widespreadness or overall LC symptom severity), PAF extraction method (max-PAF and aperiodic-adjusted PAF), and model (model 1 to model 4). No FDR correction for multiple testing was applied to these models due to exploratory nature of this analysis.

* $p < 0.05$; ** $p < 0.01$; *** $p < 0.001$.

	**Standardised β coefficient for posterior PAF [95% CI]**	**Standardised β coefficient for age [95% CI]**	**Standardised β coefficient for depression [95% CI]**	**F statistic (degrees of freedom)**	**Adj. R^2**	**Overall model p value**
**Posterior max-PAF**						
**Fatigue**						
Model 1	-0.325 [-0.73 0.08]			2.72 (1,23)	0.067	0.113
Model 2	-0.314 [-0.72 0.09]	-0.235 [-0.64 0.17]		2.11 (2,22)	0.085	0.145
Model 3	-0.282 [-0.70 0.14]		0.189 [-0.23 0.61]	1.78 (2,22)	0.061	0.191
Model 4	-0.267 [-0.68 0.16]	-0.248 [-0.65 0.16]	0.206 [-0.21 0.62]	1.76 (3,21)	0.087	0.186
**Cognitive impairments**						
Model 1	-0.191 [-0.61 0.23]			0.87 (1,23)	-0.005	0.360
Model 2	-0.194 [-0.63 0.24]	0.056 [-0.38 0.49]		0.45 (2,22)	-0.048	0.641
Model 3	-0.066 [-0.44 0.30]		**0.557**** [0.18 0.93]	5.43 (2,22)	0.270	**0.012***
Model 4	-0.067 [-0.45 0.31]	0.021 [-0.35 0.39]	**0.555**** [0.17 0.94]	3.46 (3,21)	0.235	**0.035***
**Pain duration**						
Model 1	-0.394 [-0.79 0.01]			4.23 (1,23)	0.119	0.051
Model 2	**-0.407*** [-0.80 -0.02]	0.268 [-0.12 0.66]		3.23 (2,22)	0.157	0.059
Model 3	-0.345 [-0.75 0.06]		0.218 [-0.19 0.62]	2.76 (2,22)	0.128	0.085
Model 4	-0.361 [-0.76 0.04]	0.255 [-0.13 0.64]	0.201 [-0.20 0.60]	2.53 (3,21)	0.160	0.085
**Pain widespreadness**						
Model 1	0.096 [-0.34 0.53]			0.20 (1,23)	-0.036	0.656
Model 2	0.097 [-0.35 0.55]	-0.051 [-0.50 0.40]		0.12 (2,22)	-0.082	0.883
Model 3	0.076 [-0.38 0.53]	-0.098 [-0.56 0.36]		0.20 (2,22)	-0.075	0.822
Model 4	0.078 [-0.39 0.55]	-0.036 [-0.50 0.43]	-0.092 [-0.57 0.38]	0.13 (3,21)	-0.127	0.938

**Overall symptom severity**						
Model 1	-0.311 [-0.72 0.10]			2.46 (1,23)	0.057	0.131
Model 2	-0.307 [-0.72 0.11]	-0.083 [-0.50 0.33]		1.27 (2,22)	0.022	0.301
Model 3	-0.149 [-0.44 0.14]		**0.719***** [0.43 1.01]	15.69 (2,22)	0.550	**<0.001*****
Model 4	-0.141 [-0.43 0.15]	-0.129 [-0.42 0.16]	**0.728***** [0.43 1.02]	10.70 (3,21)	0.548	**<0.001*****
**Posterior aperiodic-adjusted PAF**						
**Fatigue**						
Model 1	-0.352 [-0.76 0.05]			3.26 (1,23)	0.086	0.084
Model 2	-0.313 [-0.73 0.10]	-0.183 [-0.60 0.23]		2.03 (2,22)	0.079	0.155
Model 3	-0.329 [-0.73 0.07]		0.218 [-0.19 0.62]	2.27 (2,22)	0.096	0.127
Model 4	-0.284 [-0.70 0.13]	-0.201 [-0.62 0.21]	0.233 [-0.17 0.64]	1.85 (3,21)	0.096	0.168
**Cognitive impairments**						
Model 1	-0.166 [-0.59 0.26]			0.65 (1,23)	-0.015	0.428
Model 2	-0.185 [-0.63 0.26]	0.087 [-0.36 0.53]		0.40 (2,22)	-0.053	0.678
Model 3	-0.107 [-0.47 0.25]		**0.560**** [0.20 0.92]	5.61 (2,22)	0.278	**0.011***
Model 4	-0.116 [-0.50 0.26]	0.043 [-0.34 0.42]	**0.557**** [0.18 0.93]	3.60 (3,21)	0.245	**0.030***
**Pain duration**						
Model 1	**-0.399*** [-0.79 -0.01]			4.37 (1,23)	0.123	**0.048***
Model 2	**-0.475*** [-0.86 -0.09]	0.351 [-0.03 0.74]		4.22 (2,22)	0.211	**0.028***
Model 3	-0.372 [-0.76 0.02]		0.257 [-0.13 0.65]	3.19 (2,22)	0.154	0.061
Model 4	**-0.447*** [-0.83 -0.06]	0.333 [-0.05 0.71]	0.231 [-0.14 0.61]	3.44 (3,21)	0.234	**0.035***
**Pain widespreadness**						
Model 1	-0.044 [-0.48 0.41]			0.03 (1,22)	-0.044	0.873
Model 2	-0.026 [-0.49 0.44]	-0.044 [-0.51 0.42]		0.03 (2,21)	-0.092	0.969
Model 3	-0.043 [-0.49 0.41]		-0.117 [-0.57 0.33]	0.16 (2,21)	-0.079	0.855
Model 4	-0.038 [-0.51 0.44]	-0.024 [-0.50 0.45]	-0.113 [-0.58 0.36]	0.10 (3,20)	-0.132	0.957
**Overall symptom severity**						
Model 1	-0.313 [-0.72 0.10]			2.50 (1,23)	0.059	0.127
Model 2	-0.306 [-0.74 0.12]	-0.032 [-0.46 0.40]		1.21 (2,22)	0.017	0.317
Model 3	-0.236 [-0.51 0.04]		**0.728***** [0.45 1.00]	18.11 (2,22)	0.588	**<0.001*****
Model 4	-0.216 [-0.50 0.07]	-0.090 [-0.37 0.19]	**0.735***** [0.46 1.01]	11.91 (3,21)	0.577	**<0.001*****

Table S5. Summary of multiple linear regression models on the association between alpha band power and pain severity. Model 1 represents results of simple linear regression between dependent variable (pain severity) and independent variable (alpha band power). Models 2 and 3 outline the effects of introducing each confounding variable (age and depression, respectively); and Model 4 includes confounding effects of both age and depression. Standardised β coefficients and 95% confidence intervals (CI) for each independent variable, F statistic and degrees of freedom, adjusted R^2 and raw overall model p values are reported for each scalp region (global, anterior, central and posterior region), and model (model 1 to model 4). Only the p values from the models adjusted for both confounders (model 4) were adjusted for multiple testing using FDR correction.

	Standardised β coefficient for alpha band power [95% CI]	Standardised β coefficient for age [95% CI]	Standardised β coefficient for depression [95% CI]	F statistic (degrees of freedom)	Adj. R^2	Overall model p value	FDR-corrected p value
**Global**							
Model 1	0.063 [-0.37 0.49]			0.09 (1,23)	-0.039	0.766	---
Model 2	0.050 [-0.42 0.52]	-0.036 [-0.50 0.43]		0.06 (2,22)	-0.085	0.946	---
Model 3	0.019 [-0.40 0.44]		0.330 [-0.09 0.75]	1.37 (2,22)	0.030	0.274	---
Model 4	-0.007 [-0.47 0.45]	-0.073 [-0.53 0.38]	0.337 [-0.09 0.77]	0.92 (3,21)	-0.011	0.450	0.450
**Anterior region**							
Model 1	0.097 [-0.33 0.53]			0.22 (1,23)	-0.034	0.645	---
Model 2	0.089 [-0.37 0.55]	-0.027 [-0.49 0.43]		0.11 (2,22)	-0.080	0.895	---
Model 3	0.060 [-0.36 0.48]		0.326 [-0.09 0.74]	1.42 (2,22)	0.034	0.263	---
Model 4	0.043 [-0.41 0.49]	-0.058 [-0.51 0.39]	0.331 [-0.10 0.76]	0.93 (3,21)	-0.009	0.444	0.450
**Central region**							
Model 1	0.010 [-0.42 0.44]			<0.01 (1,23)	-0.043	0.964	---
Model 2	-0.011 [-0.48 0.46]	-0.057 [-0.53 0.41]		0.03 (2,22)	-0.088	0.968	---
Model 3	-0.026 [-0.45 0.39]		0.335 [-0.08 0.75]	1.38 (2,22)	0.031	0.273	---
Model 4	-0.060 [-0.52 0.36]	-0.093 [-0.55 0.36]	0.344 [-0.09 0.77]	0.94 (3,21)	-0.007	0.437	0.450
**Posterior region**							
Model 1	-0.016 [-0.45 0.41]			0.01 (1,23)	-0.043	0.938	---
Model 2	-0.034 [-0.49 0.42]	-0.063 [-0.52 0.40]		0.04 (2,22)	-0.087	0.958	---
Model 3	-0.040 [-0.46 0.38]		0.335 [-0.08 0.75]	1.39 (2,22)	0.031	0.270	---
Model 4	-0.065 [-0.51 0.38]	-0.089 [-0.53 0.35]	0.342 [-0.09 0.78]	0.95 (3,21)	-0.006	0.434	0.450

Exploratory pain severity-based subgroup analysis of differences in PAF and alpha power

Exploratory subgroup analysis was conducted by stratifying patients based on pain severity. These analyses were exploratory in nature and were considered hypothesis-generating. Patients were classified into moderate and severe pain subgroups based on their C19-YRS pain subscale scores. The median was used as cut-off value (median =7) (1,2),with individuals in the moderate pain group reporting pain severity scores below the median (n = 11, range of C19-YRS pain severity scores: 4–6, mean = 5.45, SD = 0.82) and individuals in the severe pain group reporting pain scores equal or above the median (n = 16, range of C19-YRS severity scores: 7–9, mean = 7.75, SD = 0.93). The group factor for the subgroup analysis models consisted of 3-levels: moderate pain group, severe pain group and control group. Peak alpha frequency derived from the two estimation methods (max-PAF and aperiodic-adjusted PAF) was entered as dependent variable in separate models. Additionally, global PAF estimates and estimates derived from each region (anterior, central and posterior) were tested separately. The models were specified as: *PAF ~ group + age.* Each model was followed by an omnibus F-test of the coefficients, which allowed to determine the overall effect of group. Significant effects of group were followed by Tukey's adjusted post-hoc pairwise comparisons.

The 3-group regression models suggested a significant effect of group on global PAF ($F(2,54) = 3.39$, $p = 0.041$ for aperiodic-adjusted PAF) and regionally in the posterior scalp area ($F(2,54) = 5.07$, $p = 0.010$ for aperiodic-adjusted PAF) (see Table S7 for max-PAF model outputs). Post-hoc pairwise comparison analysis suggested that patients reporting moderate pain presented significantly higher global and posterior PAF values compared to the healthy pain-free control group (for details of pairwise comparisons see Table S8). Moreover, additional differences localised to posterior scalp region were observed between the pain severity subgroups, particularly significantly lower PAF values in the severe pain

subgroup compared to the moderate pain subgroup ($t(54) = -2.69$, Tukey's adjusted $p = 0.025$ for aperiodic-adjusted PAF)(Figure S1). No significant differences were observed between the severe pain subgroup and pain-free controls. Subgroup comparison of PAF estimates from the anterior region yielded inconsistent observations across PAF extraction methods. A significant main effect of group was observed in association with max-PAF estimates from anterior scalp region ($F(2,54) = 4.35$, $p = 0.018$), with pairwise comparisons suggesting significantly increased anterior max-PAF in moderate pain subgroup compared to both severe pain subgroup and controls. Notwithstanding, this effect did not hold for aperiodic-adjusted PAF ($F(2,54) = 3.05$, $p = 0.056$). No differences were observed in the central region (Table S7).

Furthermore, we conducted exploratory non-parametric cluster-based permutation tests on electrode-wise data to confirm the findings of significant between-group differences from regional analysis. This approach allows for a more precise localisation of group differences across the scalp without predefining regions of interest and while controlling for multiple comparisons. Cluster-permutation testing was conducted using Fieldtrip (version 20230118) (3). First, the dependent variable was regressed on age, with residuals then used as the input for cluster analysis. Channel neighbours were identified using the function ft_prepare_neighbours based on spatial proximity between electrodes (minimum neighbourhood distance parameter set to default), with a minimum of two neighbouring electrodes for cluster formation. The analysis was implemented with 10,000 permutations to detect significant spatial clusters across electrodes (two-tailed, cluster-forming threshold and cluster significance threshold set to $\alpha = 0.05$).

Cluster-based permutation analysis comparing channel level data between moderate pain subgroup and controls showed one significant positive cluster covering occipital-parietal electrodes and extending into left frontal and central electrode sites ($p = 0.006$, cluster statistic = 80.584, cluster electrodes: Fp1, Fz, F3, FC1, C3, CP1, P3, O2, P4, P8, CP2, AF7, AF3, AFz, F1, FC3, CP3, P1, P5, PO7, PO3, POz, PO4, PO8, P6, P2, CP4 for aperiodic-

adjusted PAF)(Figure S2.B; bottom left panel). The results indicate electrodes sites where PAF was significantly increased in patients with moderate pain as compared to pain-free controls. Additionally, the topographies differed between patient subgroups, suggesting a significant decrease in PAF over occipital-parietal electrodes extending into central and left temporal electrode sites ($p = 0.005$, cluster statistic = -77.491, cluster electrodes: Fz, F3, FC1, C3, CP1, O2, P4, P8, CP6, CP2, Cz, C4, FC2, F4, F1, FC3, C1, CP3, PO4, PO8, P6, P2, CP4, C2, FC4, F2 for aperiodic-adjusted PAF) in patients with severe pain compared to the moderate pain subgroup (Figure S2.B; top right panel). No significant differences in PAF were observed between the severe pain patient subgroup and healthy controls (Figure S2.B; bottom right panel). Please refer to Figure S3 legend for description of results of cluster-based permutation analysis on max-PAF estimates.

No significant differences in alpha power were observed between moderate and severe pain subgroups and pain-free controls (see Table S9 for detailed omnibus test results).

Table S6. Summary of demographic and clinical variables for Long COVID patients in the moderate pain subgroup and severe pain subgroup. Subgroups were defined based on median pain severity (median = 7) with a moderate pain subgroup including patients scoring below median value on the C19-YRS pain subscale (n = 11, range of pain severity scores: 4–6), and a severe pain subgroup including patients scoring equal or above median value (n = 16, range of pain severity scores: 7–9).

	**Severe pain subgroup (n = 16)**	**Moderate pain subgroup (n = 11)**
**Demographic characteristics**		
Age, mean (SD), years	42.9 (10.2)	50.2 (12.1)
Sex, n (%)		
female	10 (62.5)	7 (63.6)
male	6 (37.5)	4 (36.4)
Ethnicity, n (%)		
white	14 (87.5)	9 (81.8)
asian	2 (12.5)	2 (18.2)
black	0 (0.0)	0.0 (0.0)
mixed	0 (0.0)	0.0 (0.0)
other	0 (0.0)	0 (0.0)
BMI, mean (SD), kg/m^2	31.8 (1.5) (missing: 2)	31.8 (9.2) (missing: 2)
Smoking status, n (%)		(missing: 1)
none/former	15 (93.7)	10 (100.0)
active	1 (6.3)	0 (0.0)
**Psychological variables**		
PHQ-9 scores, mean (SD)	13.2 (6.0)	12.0 (4.1) (missing: 2)
**Clinical characteristics**		
Duration of LC symptoms, mean (SD)	15.5 (7.9)	14.7 (5.7)
Number of COVID-19 infections, n (%)	(missing: 1)	
infected once	10 (66.7)	3 (27.3)
infected twice	5 (33.3)	5 (45.4)
infected 3 times	0 (0.0)	3 (27.3)
Pre-exiting comorbidities (prior LC), n (%)	(missing: 4)	
type-1 or type-2 diabetes mellitus	0 (0.0)	0 (0.0)
Hypertension	0 (0.0)	0 (0.0)
chronic respiratory conditions	1 (8.3)	2 (18.2)
chronic heart disease	0 (0.0)	1 (9.1)
chronic neurological conditions	0 (0.0)	1 (9.1)
chronic musculoskeletal conditions	0 (0.0)	2 (18.2)
chronic kidney disease	0 (0.0)	0 (0.0)
chronic liver disease	0 (0.0)	0 (0.0)
no pre-existing medical conditions	3 (25.0)	3 (27.3)
other	9 (75.0)	5 (45.4)
Medication, n (%)		
Paracetamol	9 (56.2)	9 (81.8)
NSAIDs	5 (31.2)	8 (72.7)
Opioids	2 (12.5)	2 (18.2)
Gabapentinoids	2 (12.5)	0 (0.0)
SSRIs	0 (0.0)	3 (27.3)
SNRIs	0 (0.0)	0 (0.0)
Tricyclic antidepressants	0 (0.0)	2 (18.2)
Antihistamines	1 (6.2)	1 (9.1)
Beta-blockers	1 (6.2)	1 (9.1)
H_2 receptor antagonists	2 (12.5)	0 (0.0)
Proton-pump inhibitors	2 (12.5)	0 (0.0)
No medication	3 (18.7)	1 (9.1)
**Long COVID symptoms**		
C19-YRS items related to neurological and psychological symptoms of LC		

Fatigue	8.6 (1.3)	7.6 (1.4)
Pain	7.7 (0.9)	5.4 (0.8)
Cognition	6.6 (2.5)	6.2 (2.2)
Anxiety	6.3 (2.5)	5.8 (2.6)
Depression	5.7 (2.0)	6.7 (2.2)
Overall symptom severity, mean (SD)	46.7 (18.6)	42.7 (16.1)

Table S7. Summary of omnibus test on multiple linear regression models testing group differences in peak alpha frequency (PAF), adjusted for the effects of age. Two-group comparison (LC patient group and healthy pain-free control group) and 3-group comparison (moderate pain subgroup, severe pain subgroup and healthy pain-free control group) were tested on separate models. F statistic (F), degrees of freedom (df) and p values are reported for each independent variable in the model. Results relevant to each PAF extraction method (max-PAF and aperiodic-adjusted PAF) and each scalp region (global, anterior, central and posterior region) are presented in the table. * p < 0.05; ** p < 0.01.

Model	Group			Age		
	F	df	p value	F	df	p value
**Global**						
**Max-PAF models**						
2-group model	1.56	1,59	0.217	0.24	1,59	0.626
3-group model	3.42	2,54	**0.040***	0.89	1,54	0.350
**Aperiodic adjusted PAF-models**						
2-group model	2.60	1,59	0.112	0.20	1,59	0.654
3-group model	3.39	2,54	**0.041***	0.72	1,54	0.401
**Anterior region**						
**Max-PAF models**						
2-group model	3.62	1,59	0.062	0.01	1,59	0.925
3-group model	4.35	2,54	**0.018***	0.20	1,54	0.658
**Aperiodic adjusted PAF-models**						
2-group model	3.88	1,59	0.054	0.25	1,59	0.619
3-group model	3.05	2,54	0.056	0.53	1,54	0.470
**Central region**						
**Max-PAF models**						
2-group model	0.90	1,59	0.345	0.18	1,59	0.674
3-group model	2.98	2,54	0.059	0.74	1,54	0.393
**Aperiodic adjusted PAF-models**						
2-group model	1.09	1,59	0.300	0.26	1,59	0.612
3-group model	2.56	2,54	0.087	0.89	1,54	0.349
**Posterior region**						
**Max-PAF models**						
2-group model	3.05	1,59	0.086	0.05	1,59	0.823
3-group model	5.07	2,54	**0.010****	0.42	1,54	0.519
**Aperiodic adjusted PAF-models**						
2-group model	3.65	1,59	0.061	0.07	1,59	0.787
3-group model	5.07	2,54	**0.010****	<0.01	1,54	0.975

Table S8. Post-hoc pairwise comparisons of peak alpha frequency (PAF) between groups, adjusted for the effects of age. Only post-hoc pairwise comparisons from regression models showing a significant effect of group on omnibus test are presented in the table. Mean and standard deviation (SD) for each group, adjusted mean difference and 95% confidence intervals (CI), standard error (SE), t values and degrees of freedom (df), and p values are reported for each pairwise comparison. Note that p values for the 3-group model (marked with †) are adjusted with Tukey method for multiple testing. * $p < 0.05$; ** $p < 0.01$.

**Model**	**Comparison (group 1 vs group 2)**	**Group 1 mean ± SD**	**Group 2 mean ± SD**	**Adjusted mean difference [95% CI]**	**SE**	**t value (df)**	**p value**
			**Global**				
**Max-PAF models**							
3-group model	Moderate pain subgroup – healthy pain-free controls	10.61 (±1.23)	9.76 (±0.99)	0.891 [0.01 1.78]	0.37	2.42 (54)	**0.049*** †
	Severe pain subgroup – healthy pain-free controls	9.71 (±0.99)	9.76 (±0.99)	-0.095 [-0.88 0.69]	0.32	-0.29 (54)	0.954 †
	Severe pain subgroup – moderate pain subgroup	9.71 (±0.99)	10.61 (±1.23)	-0.986 [-1.99 0.02]	0.42	-2.36 (54)	0.057 †
**Aperiodic-adjusted PAF models**							
3-group model	Moderate pain subgroup – healthy pain-free controls	10.59 (±0.96)	9.84 (±0.92)	0.783 [0.02 1.55]	0.32	2.46 (54)	**0.044*** †
	Severe pain subgroup – healthy pain-free controls	9.83 (±0.79)	9.84 (±0.92)	-0.037 [-0.71 0.64]	0.28	-0.13 (54)	0.990 †
	Severe pain subgroup – moderate pain subgroup	9.83 (±0.79)	10.59 (±0.96)	-0.820 [-1.69 0.05]	0.36	-2.27 (54)	0.069 †
			**Anterior region**				
**Max-PAF models**							
3-group model	Moderate pain subgroup – healthy pain-free controls	10.55 (±1.25)	9.53 (±0.95)	1.039 [0.17 1.91]	0.32	2.87 (54)	**0.016*** †
	Severe pain subgroup – healthy pain-free controls	9.59 (±0.96)	9.53 (±0.95)	0.044 [-0.72 0.81]	0.32	0.14 (54)	0.990 †
	Severe pain subgroup – moderate pain subgroup	9.59 (±0.96)	10.55 (±1.25)	-0.996 [-1.99 -0.01]	0.41	-2.42 (54)	**0.049*** †
			**Posterior region**				
**Max-PAF models**							
3-group model	Moderate pain subgroup – healthy pain-free controls	10.98 (±1.16)	9.91 (±1.10)	1.118 [0.23 2.01]	0.37	3.02 (54)	**0.011*** †
	Severe pain subgroup – healthy pain-free controls	9.87 (±0.84)	9.91 (±1.10)	-0.046 [-0.83 0.74]	0.33	-0.14 (54)	0.989 †
	Severe pain subgroup – moderate pain subgroup	9.87 (± 0.84)	10.98 (±1.16)	-1.164 [-2.18 -0.15]	0.42	-2.77 (54)	**0.021*** †
**Aperiodic-adjusted PAF models**							
3-group model	Moderate pain subgroup – healthy pain-free controls	10.90 (±0.97)	9.99 (±0.83)	0.913 [0.19 1.63]	0.30	3.06 (54)	**0.009**** †
	Severe pain subgroup – healthy pain-free controls	9.99 (±0.74)	9.99 (±0.83)	0.001 [-0.63 0.63]	0.26	<0.01 (54)	1.000 †
	Severe pain subgroup – moderate pain subgroup	9.99 (±0.74)	10.90 (±0.97)	-0.912 [-1.73 -0.10]	0.34	-2.69 (54)	**0.025*** †

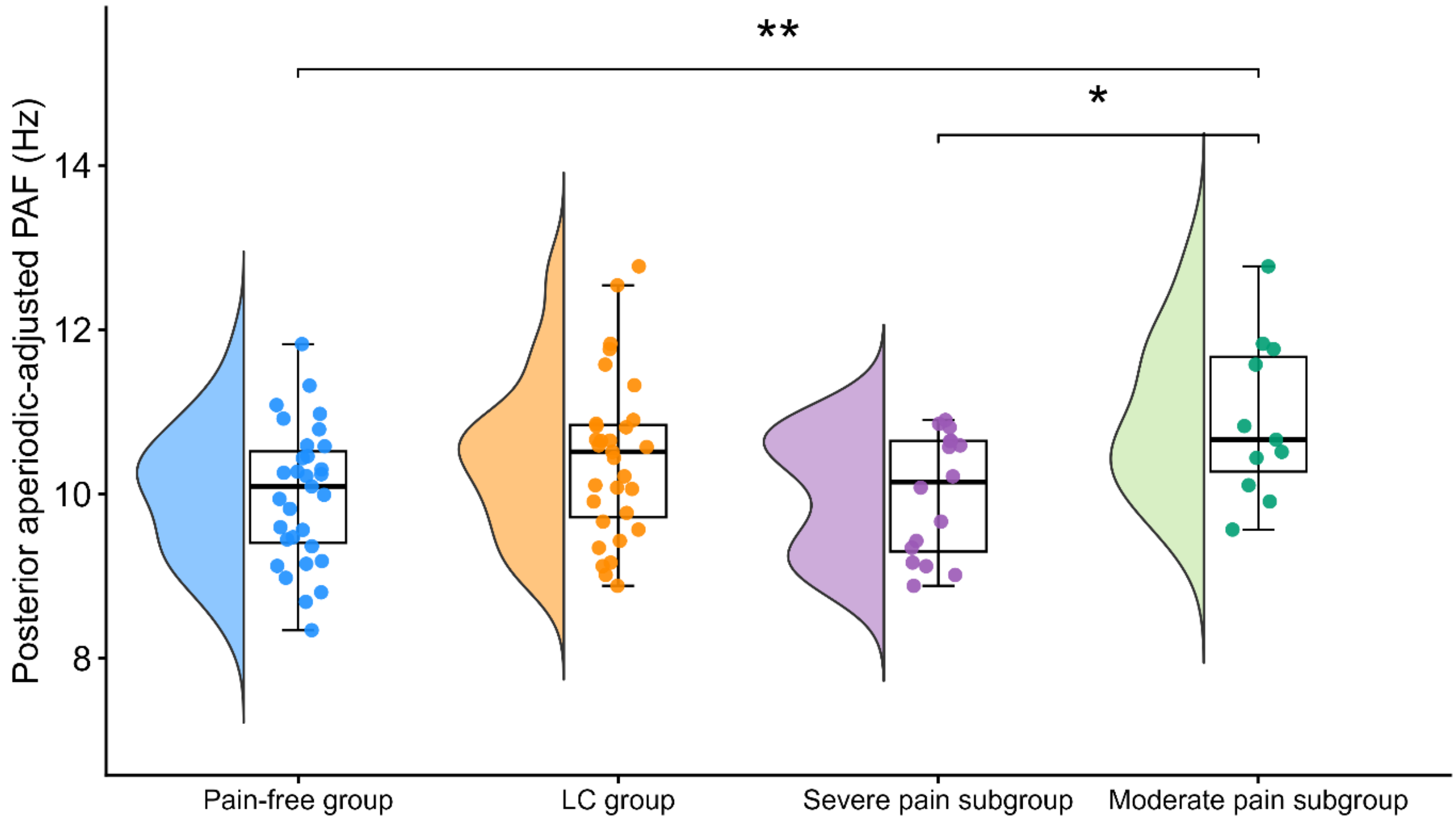

Figure S1. Comparison of peak alpha frequency (PAF) estimates over the posterior scalp region between LC patient group (n = 31) and healthy pain-free controls (n = 31). LC patient group is further stratified into pain severity-based subgroups: moderate pain subgroup (n = 11) and a severe pain subgroup (n=16). Data is shown in raincloud plots combining un-mirrored violin plots, boxplots and individual data points to provide a comprehensive view of the distribution and spread of the data for each group and subgroup. Data represented in the figure is derived from aperiodic-adjusted PAF estimates. p values were obtained from age-adjusted regression models. * $p < 0.05$; ** $p < 0.01$.

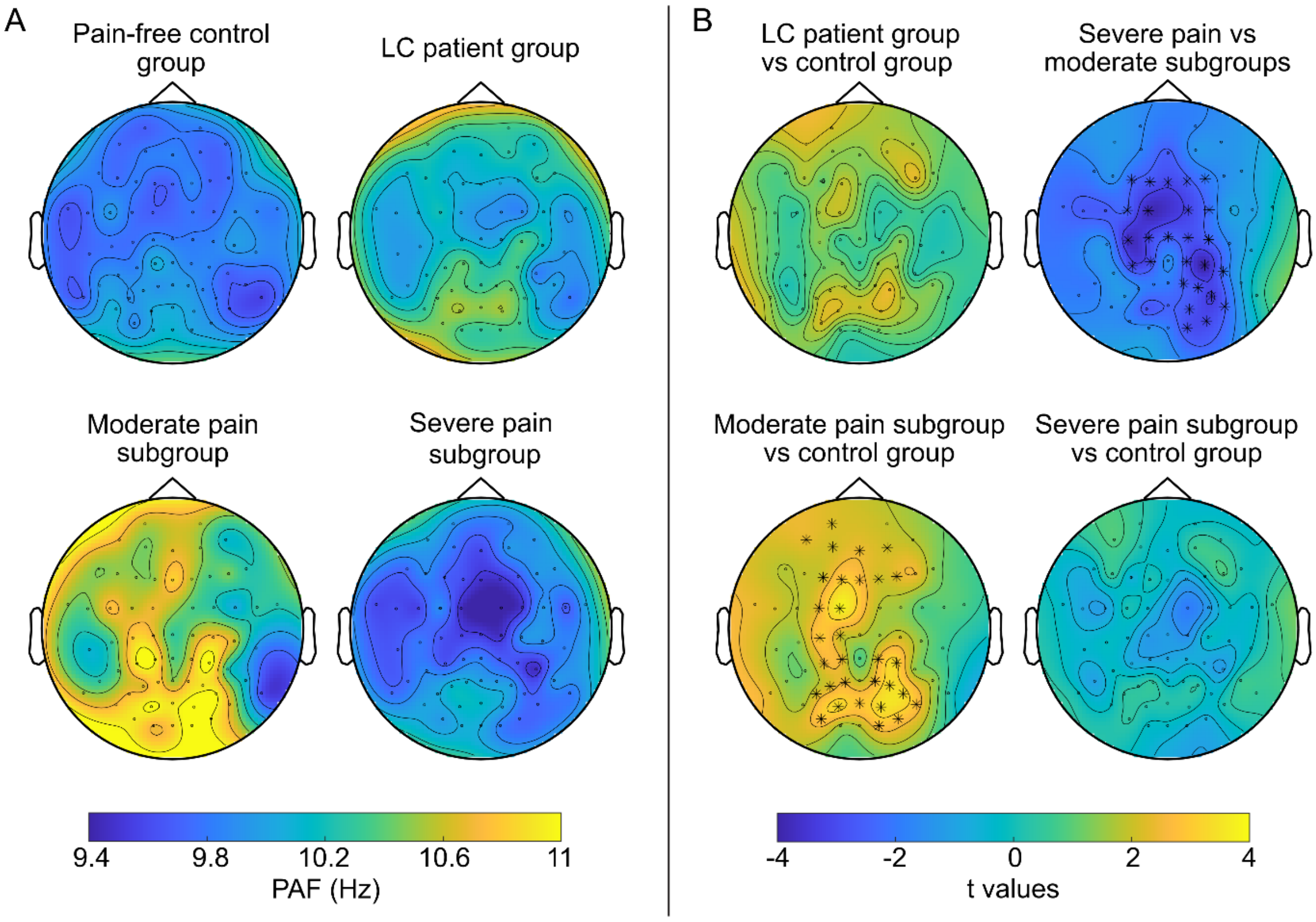

Figure S2. Cluster-based permutation comparison of peak alpha frequency (PAF) between groups at channel level. (A) Topographical plots of PAF extracted for each electrode and averaged across participants within the healthy pain-free control group (n = 31), Long COVID (LC) patient group (n = 31), LC patient moderate pain subgroup (n = 11), and LC patient severe pain subgroup (n = 16). (B) Channel-level comparison of PAF estimates between groups. Scalp maps represent topographical distribution of t-values for the comparison between groups, with warmer and colder colours representing increased and decreased PAF, respectively. Asterisks (*) denote electrode sites identified as part of significant clusters at significance level $p < 0.05$. Top left plot shows comparison between LC patient group and control group, with no significant clusters found. Top right plot shows comparison between severe pain and moderate pain LC patient subgroups, highlighting one significant negative cluster concentrated over central to centro-parietal electrodes and extending into frontal and right-lateralised occipital-parietal electrode sites. Bottom left panel shows comparison between patients reporting moderate pain and controls, highlighting one

significant positive cluster covering occipital-parietal electrodes and extending into left frontal and central electrode sites. Bottom right panel shows comparison between patients reporting severe pain and controls, with no significant clusters found. Data represented in the figure is derived from aperiodic-adjusted PAF estimates.

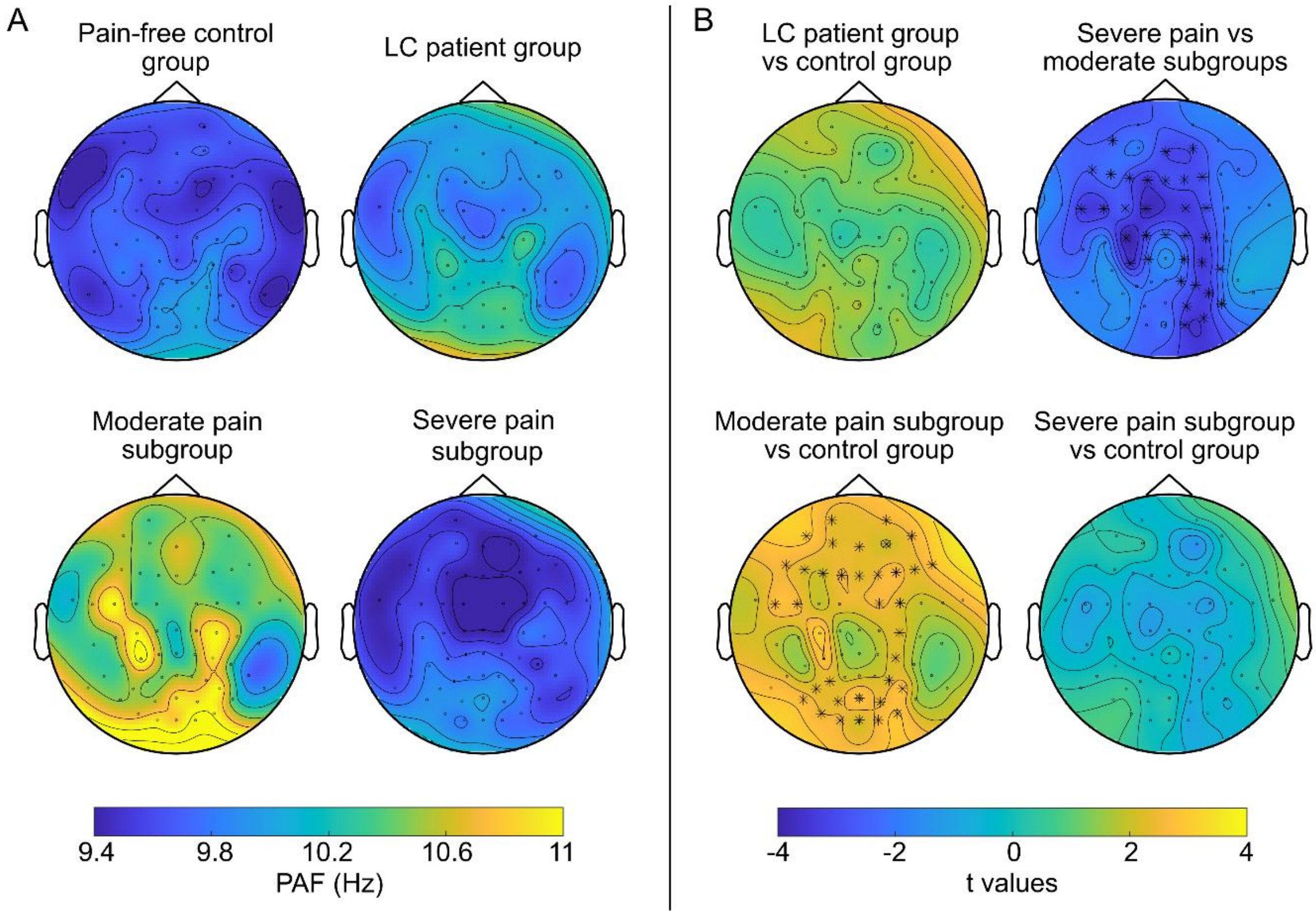

Figure S3. Cluster-based permutation comparison of max-peak alpha frequency (max-PAF) between groups at channel level. A) Topographical plots of PAF extracted for each electrode and averaged across participants within the healthy pain-free control group (n = 31), Long COVID (LC) patient group (n = 31), LC patient moderate pain subgroup (n = 11), and LC patient severe pain subgroup (n = 16). B) Channel-level comparison of PAF estimates between groups. Scalp maps represent topographical distribution of t-values for the comparison between groups, with warmer and colder colours representing increased and decreased PAF, respectively. Asterisks (*) denote electrode sites identified as part of significant clusters at significance level $p < 0.05$. Top left plot shows comparison between LC patient group and control group, with no significant clusters found. Top right plot shows comparison between severe pain and moderate pain LC patient subgroups, highlighting one significant negative cluster concentrated over frontal and central areas and extending into right-lateralised occipital-parietal electrode sites ($p = 0.005$, cluster statistic = -92.532). Bottom left panel shows comparison between patients reporting moderate pain and controls,

highlighting one significant positive cluster covering bilateral frontal, right central and bilateral occipital-parietal regions ($p = 0.005$, cluster statistic = 87.134). Bottom right panel shows comparison between patients reporting severe pain and controls, with no significant clusters found. Data represented in the figure is derived from max-PAF estimates.

Table S9. Summary of omnibus test on multiple linear regression models testing group differences in alpha band power, adjusted for the effects of age. Two-group comparison (LC patient group and healthy pain-free control group) and 3-group comparison (moderate pain subgroup, severe pain subgroup and healthy pain-free control group) were tested on separate models. F statistic (F), degrees of freedom (df) and p values are reported for each independent variable in the model. Results relevant to global estimates and each tested scalp region (anterior, central and posterior region) are presented in the table.

Model	Group			Age		
	F	df	p value	F	df	p value
**Global**						
2-group model	0.05	1,59	0.821	0.85	1,59	0.361
3-group model	0.13	2,54	0.882	1.12	1,54	0.295
**Anterior region**						
2-group model	0.01	1,59	0.932	1.47	1,59	0.231
3-group model	0.15	2,54	0.863	1.83	1,54	0.182
**Central region**						
2-group model	0.09	1,59	0.760	0.21	1,59	0.645
3-group model	0.05	2,54	0.947	0.52	1,54	0.475
**Posterior region**						
2-group model	0.04	1,59	0.832	0.98	1,59	0.327
3-group model	0.11	2,54	0.899	1.02	1,54	0.316

Sensitivity analysis of group differences in peak alpha frequency including adjustment for the effects of depression

Depression-adjusted between-group differences in PAF were tested with multiple linear regression models including a group factor and covariates age and depression. The group factor for the 2-group comparison consisted of 2-levels: LC patients vs controls; while the subgroup analysis models consisted of 3-levels: moderate pain group, severe pain group and control group. The models were specified as: *PAF ~ group + age + depression score.* Peak alpha frequency derived from the two estimation methods (max-PAF and aperiodic-adjusted PAF) for global and each tested scalp region (anterior, central, posterior) was entered as dependent variable in separate models. Each model was followed by an omnibus F-test of the coefficients which allowed to determine the overall effect of group, and subsequent Tukey's adjusted post-hoc pairwise comparisons where group effects were significant (for details of omnibus test outputs and pairwise comparisons see Table S10 and Table S11, respectively).

Results of regression analysis testing depression-adjusted differences between groups were largely inconsistent across PAF estimation methods. When comparing overall LC patient group and controls, a significant main effect of group and depression was found for max-PAF estimates only, and not for aperiodic-adjusted PAF, at global and anterior region level; with post-hoc comparisons suggesting increased global and anterior max-PAF values in patients compared to controls. At subgroup level, a significant effect of group on PAF was found globally and in posterior scalp region, consistently for both max-PAF and aperiodic-adjusted PAF. Subsequent post-hoc pairwise comparisons showed increased global PAF in moderate pain subgroup compared to controls. In regard to posterior region, we observed significantly increased PAF in the moderate pain subgroup compared to the severe pain subgroup, which held consistently for both PAF extraction methods. Only the max-PAF model survived Tukey-adjusted pairwise comparison testing with respect to comparison between moderate pain

subgroup and controls, highlighting significantly increased PAF in moderate pain subgroup compared to controls. A significant effect of group was also found in association with anterior and central max-PAF estimates, with moderate pain subgroup showing increased PAF compared to controls but no differences between pain severity subgroups.

Table S10. Summary of omnibus test on multiple linear regression models testing group differences in peak alpha frequency (PAF), adjusted for the effects of age and depression. Two-group comparison (LC patient group and healthy pain-free control group) and 3-group comparison (moderate pain subgroup, severe pain subgroup and healthy pain-free control group) were tested on separate models. F statistic (F), degrees of freedom (df) and p values are reported for each independent variable in the model. Results relevant to each PAF extraction method (max-PAF and aperiodic-adjusted PAF) and each scalp region (global, anterior, central and posterior region) are presented in the table. * p < 0.05.

Model	Group			Age			Depression		
	**F**	**df**	**p value**	**F**	**df**	**p value**	**F**	**df**	**p value**
**Global**									
**Max-PAF models**									
2-group model	4.49	1,52	**0.039***	1.05	1,52	0.310	4.11	1,52	**0.048***
3-group model	4.42	2,51	**0.017***	1.69	1,51	0.199	3.69	1,51	0.060
**Aperiodic adjusted PAF-models**									
2-group model	2.80	1,52	0.100	0.73	1,52	0.396	1.88	1,52	0.176
3-group model	3.32	2,51	**0.044***	1.24	1,51	0.271	1.56	1,51	0.217
**Anterior region**									
**Max-PAF models**									
2-group model	7.11	1,52	**0.010***	0.40	1,52	0.530	5.38	1,52	**0.024***
3-group model	5.85	2,51	**0.005***	0.82	1,51	0.370	4.93	1,51	**0.031***
**Aperiodic adjusted PAF-models**									
2-group model	2.95	1,52	0.092	0.66	1,52	0.418	1.36	1,52	0.249
3-group model	2.64	2,51	0.081	1.02	1,51	0.318	1.12	1,51	0.295
**Central region**									
**Max-PAF models**									
2-group model	3.78	1,52	0.057	0.81	1,52	0.372	3.86	1,52	0.055
3-group model	3.96	2,51	**0.025***	1.37	1,51	0.248	3.45	1,51	0.069
**Aperiodic adjusted PAF-models**									
2-group model	2.11	1,52	0.152	0.80	1,52	0.376	1.95	1,52	0.168
3-group model	2.81	2,51	0.069	1.30	1,51	0.260	1.63	1,51	0.207
**Posterior region**									
**Max-PAF models**									
2-group model	2.01	1,52	0.162	0.14	1,52	0.711	0.61	1,52	0.436
3-group model	4.43	2,51	**0.017***	0.51	1,51	0.479	0.38	1,51	0.538
**Aperiodic adjusted PAF-models**									
2-group model	0.96	1,52	0.333	0.11	1,52	0.746	0.01	1,52	0.920
3-group model	3.90	2,51	**0.026***	<0.01	1,51	0.990	0.01	1,51	0.922

Table S11. Post-hoc pairwise comparisons of peak alpha frequency (PAF) between groups, adjusted for the effects of age and depression. Only post-hoc pairwise comparisons from regression models showing a significant effect of group on omnibus test are presented in the table. Mean and standard deviation (SD) for each group, adjusted mean difference and 95% confidence intervals (CI), standard error (SE), t values and degrees of freedom (df), and p values are reported for each pairwise comparison. Note that p values for the 3-group model (marked with †) are adjusted with Tukey method for multiple testing. * p < 0.05; ** p < 0.01.

Model	Comparison (group 1 vs group 2)	Group 1 mean ± SD	Group 2 mean ± SD	Adjusted mean difference [95% CI]	SE	t value (df)	p value
**Global**							
**Max-PAF models**							
2-group model	All patients – healthy pain-free controls	10.11 (±1.15)	9.76 (±0.99)	0.950 [0.05 1.85]	0.45	2.12 (52)	**0.039***
3-group model	Moderate pain subgroup – healthy pain-free controls	10.61 (±1.23)	9.76 (±0.99)	1.445 [0.24 2.65]	0.50	2.89 (51)	**0.015*** †
	Severe pain subgroup – healthy pain-free controls	9.71 (±0.99)	9.76 (±0.99)	0.576 [-0.57 1.72]	0.47	1.22 (51)	0.449 †
	Severe pain subgroup – moderate pain subgroup	9.71 (±0.99)	10.61 (±1.23)	-0.869 [-1.91 0.17]	0.43	-2.02 (51)	0.118 †
**Aperiodic-adjusted PAF models**							
3-group model	Moderate pain subgroup – healthy pain-free controls	10.59 (±0.96)	9.84 (±0.92)	1.070 [0.01 2.13]	0.44	2.44 (51)	**0.047*** †
	Severe pain subgroup – healthy pain-free controls	9.83 (±0.79)	9.84 (±0.92)	0.344 [-0.66 1.35]	0.41	0.83 (51)	0.687 †
	Severe pain subgroup – moderate pain subgroup	9.83 (±0.79)	10.59 (±0.96)	-0.726 [-1.64 0.19]	0.38	-1.92 (51)	0.143 †
**Anterior region**							
**Max-PAF models**							
2-group model	All patients – healthy pain-free controls	10.06 (±1.22)	9.53 (±0.95)	1.160 [0.29 2.03]	0.43	2.67 (52)	**0.010***
3-group model	Moderate pain subgroup – healthy pain-free controls	10.55 (±1.25)	9.53 (±0.95)	1.640 [0.47 2.81]	0.48	3.39 (51)	**0.004**** †
	Severe pain subgroup – healthy pain-free controls	9.59 (±0.96)	9.53 (±0.95)	0.791 [-0.31 1.90]	0.46	1.73 (51)	0.204 †
	Severe pain subgroup – moderate pain subgroup	9.59 (±0.96)	10.55 (±1.25)	-0.849 [-1.85 0.16]	0.42	-2.04 (51)	0.113 †
**Central region**							
**Max-PAF models**							
3-group model	Moderate pain subgroup – healthy pain-free controls	10.59 (±1.20)	9.81 (±1.04)	1.394 [0.15 2.63]	0.51	2.71 (51)	**0.024*** †
	Severe pain subgroup – healthy pain-free controls	9.70 (±1.00)	9.81 (±1.04)	0.517 [-0.66 1.69]	0.49	1.06 (51)	0.541 †
	Severe pain subgroup – moderate pain subgroup	9.70 (±1.00)	10.59 (±1.20)	-0.876 [-1.94 0.19]	0.44	-1.98 (51)	0.127 †
**Posterior region**							
**Max-PAF models**							
3-group model	Moderate pain subgroup – healthy pain-free controls	10.98 (±1.16)	9.91 (±1.10)	0.747 [0.01 1.48]	0.30	2.56 (51)	**0.045*** †
	Severe pain subgroup – healthy pain-free controls	9.87 (±0.84)	9.91 (±1.10)	-0.449 [-1.52 0.62]	0.44	0.36 (51)	0.571 †
	Severe pain subgroup – moderate pain subgroup	9.87 (± 0.84)	10.98 (±1.16)	-1.196 [-2.32 -0.07]	0.47	-2.57 (51)	**0.035*** †

**Aperiodic-adjusted PAF models**							
3-group model	Moderate pain subgroup – healthy pain-free controls	10.90 (±0.97)	9.99 (±0.83)	0.901 [-0.10 1.90]	0.41	2.17 (51)	0.086 †
	Severe pain subgroup – healthy pain-free controls	9.99 (±0.74)	9.99 (±0.83)	-0.027 [-0.98 0.92]	0.39	-0.027 (51)	0.836 †
	Severe pain subgroup – moderate pain subgroup	9.99 (±0.74)	10.90 (±0.97)	-1.791 [-0.06 -2.60]	0.36	-2.60 (51)	**0.032*** †

Exploratory analysis of association between pain severity and absolute spectral power of delta, theta and beta bands

Table S12. Summary of multiple linear regression models on the association between absolute spectral power of canonical EEG frequency bands and pain severity. Model 1 represents results of simple linear regression between dependent variable (pain severity) and independent variable (absolute spectral power of each canonical EEG frequency band: delta, theta and beta). Models 2 and 3 outline the effects of introducing each confounding variable (age and depression, respectively); and Model 4 includes confounding effects of both age and depression. Standardised β coefficients and 95% confidence intervals (CI) for each independent variable, F statistic and degrees of freedom, adjusted R^2 and raw overall model p values are reported for each combination of EEG frequency band (delta, theta and beta), scalp region (global, anterior, central and posterior region), and model (model 1 to model 4).

	Standardised β coefficient for spectral power [95% CI]	Standardised β coefficient for age [95% CI]	Standardised β coefficient for depression [95% CI]	F statistic (degrees of freedom)	Adj. R^2	Overall model p value
**Global**						
**Delta band power**						
Model 1	<0.001 [-0.43 0.43]			<0.01 (1,23)	-0.043	0.999
Model 2	-0.045 [-0.58 0.49]	-0.079 [-0.63 0.46]		0.05 (2,22)	-0.086	0.955
Model 3	-0.049 [-0.47 0.37]		0.340 [-0.08 0.76]	1.40 (2,22)	0.032	0.267
Model 4	-0.139 [-0.67 0.39]	-0.151 [-0.67 0.37]	0.361 [-0.07 0.79]	1.03 (3,21)	0.003	0.401
**Theta band power**						
Model 1	0.342 [-0.06 0.75]			3.04 (1,23)	0.078	0.094
Model 2	0.340 [-0.08 0.76]	-0.011 [-0.43 0.41]		1.46 (2,22)	0.037	0.255
Model 3	0.257 [-0.17 0.69]		0.243 [-0.19 0.67]	2.23 (2,22)	0.093	0.131
Model 4	0.251 [-0.19 0.70]	-0.035 [-0.45 0.38]	0.247 [-0.20 0.69]	1.43 (3,21)	0.051	0.262
**Beta band power**						
Model 1	0.071 [-0.36 0.42]			0.12 (1,23)	-0.038	0.735
Model 2	0.065 [-0.38 0.51]	-0.044 [-0.49 0.40]		0.08 (2,22)	-0.083	0.926
Model 3	0.159 [-0.26 0.58]		0.371 [-0.05 0.79]	1.71 (2,22)	0.056	0.204
Model 4	0.152 [-0.29 0.59]	-0.050 [-0.48 0.38]	0.372 [-0.06 0.81]	1.11 (3,21)	0.014	0.366
**Anterior region**						
**Delta band power**						
Model 1	0.050 [-0.38 0.48]			0.06 (1,23)	-0.041	0.810
Model 2	0.028 [-0.54 0.59]	-0.035 [-0.60 0.53]		0.04 (2,22)	-0.087	0.964
Model 3	-0.039 [-0.47 0.39]		0.343 [-0.09 0.77]	1.39 (2,22)	0.031	0.270
Model 4	-0.155 [-0.74 0.43]	-0.169 [-0.73 0.40]	0.382 [-0.08 0.84]	1.03 (3,21)	0.004	0.400
**Theta band power**						

Model 1	0.347 [-0.06 0.75]			3.15 (1,23)	0.082	0.089
Model 2	0.345 [-0.07 0.76]	-0.027 [-0.44 0.39]		1.52 (2,22)	0.041	0.241
Model 3	0.263 [-0.17 0.69]		0.240 [-0.19 0.67]	2.28 (2,22)	0.096	0.126
Model 4	0.258 [-0.18 0.70]	-0.046 [-0.46 0.37]	0.245 [-0.20 0.69]	1.47 (3,21)	0.055	0.251
**Beta band power**						
Model 1	0.107 [-0.32 0.53]			0.26 (1,23)	-0.032	0.612
Model 2	0.100 [-0.35 0.55]	-0.035 [-0.48 0.41]		0.14 (2,22)	-0.077	0.870
Model 3	0.200 [-0.22 0.63]		0.382 [-0.04 0.80]	1.91 (2,22)	0.071	0.171
Model 4	0.193 [-0.24 0.63]	-0.039 [-0.46 0.39]	0.382 [-0.05 0.81]	1.23 (3,21)	0.028	0.323
**Central region**						
**Delta band power**						
Model 1	0.030 [-0.40 0.46]			0.02 (1,23)	-0.043	0.888
Model 2	0.004 [-0.51 0.51]	-0.051 [-0.56 0.46]		0.03 (2,22)	-0.088	0.969
Model 3	-0.012 [-0.43 0.41]		0.334 [-0.09 0.75]	1.37 (2,22)	0.030	0.275
Model 4	-0.066 [-0.57 0.43]	-0.104 [-0.60 0.39]	0.346 [-0.09 0.78]	0.94 (3,21)	-0.007	0.437
**Theta band power**						
Model 1	0.329 [-0.08 0.74]			2.80 (1,23)	0.070	0.108
Model 2	0.328 [-0.09 0.75]	-0.015 [-0.44 0.40]		1.34 (2,22)	0.028	0.282
Model 3	0.250 [-0.17 0.67]		0.255 [-0.17 0.68]	2.21 (2,22)	0.091	0.134
Model 4	0.244 [-0.19 0.68]	-0.038 [-0.46 0.38]	0.258 [-0.18 0.70]	1.42 (3,21)	0.050	0.265
**Beta band power**						
Model 1	0.037 [-0.39 0.42]			0.03 (1,23)	-0.042	0.861
Model 2	0.035 [-0.41 0.48]	-0.052 [-0.49 0.39]		0.04 (2,22)	-0.086	0.956
Model 3	0.138 [-0.29 0.57]		0.370 [-0.06 0.80]	1.62 (2,22)	0.049	0.221
Model 4	0.136 [-0.30 0.58]	-0.069 [-0.49 0.35]	0.373 [-0.07 0.81]	1.07 (3,21)	0.009	0.382
**Posterior region**						
**Delta band power**						
Model 1	-0.018 [-0.45 0.41]			0.01 (1,23)	-0.043	0.932
Model 2	-0.080 [-0.64 0.48]	-0.102 [-0.68 0.45]		0.08 (2,22)	-0.083	0.927
Model 3	-0.076 [-0.50 0.34]		0.345 [-0.08 0.78]	1.45 (2,22)	0.036	0.257
Model 4	-0.200 [-0.75 0.35]	-0.195 [-0.74 0.35]	0.377 [-0.06 0.81]	1.13 (3,21)	0.016	0.359
**Theta band power**						
Model 1	0.339 [-0.07 0.74]			2.99 (1,23)	0.077	0.097
Model 2	0.338 [-0.08 0.76]	-0.011 [-0.43 0.41]		1.43 (2,22)	0.035	0.260
Model 3	0.256 [-0.17 0.68]		0.246 [-0.18 0.67]	2.23 (2,22)	0.093	0.131
Model 4	0.250 [-0.19 0.69]	-0.035 [-0.45 0.38]	0.250 [-0.19 0.69]	1.43 (3,21)	0.051	0.261
**Beta band power**						
Model 1	0.023 [-0.41 0.45]			0.01 (1,23)	-0.043	0.911
Model 2	0.010 [-0.45 0.47]	-0.050 [-0.51 0.41]		0.03 (2,22)	-0.088	0.968
Model 3	0.083 [-0.34 0.50]		0.347 [-0.07 0.77]	1.46 (2,22)	0.037	0.253
Model 4	0.069 [-0.38 0.52]	-0.053 [-0.49 0.39]	0.347 [-0.08 0.78]	0.95 (3,21)	-0.006	0.432

Exploratory analysis of differences in absolute spectral power of delta, theta and beta bands between patients and controls

Table S13. Summary of omnibus test on multiple linear regression models testing group differences in absolute band power of canonical EEG frequency bands, adjusted for the effects of age. Two-group comparison (LC patient group and healthy pain-free control group) and 3-group comparison (moderate pain subgroup, severe pain subgroup and healthy pain-free control group) were tested on separate models. F statistic (F), degrees of freedom (df) and p values are reported for each independent variable in the model. Results relevant to each EEG frequency band (delta, theta and beta) and each tested scalp region (global, anterior, central and posterior region) are presented in the table.

Model	Group			Age		
	F	df	p value	F	df	p value
**Global**						
**Delta band power**						
2-group model	0.03	1,59	0.870	1.71	1,59	0.197
3-group model	0.01	2,54	0.988	1.73	1,54	0.194
**Theta band power**						
2-group model	0.08	1,59	0.782	1.10	1,59	0.298
3-group model	0.74	2,54	0.482	1.14	1,54	0.289
**Beta band power**						
2-group model	0.01	1,59	0.937	1.50	1,59	0.226
3-group model	0.16	2,54	0.851	1.32	1,54	0.256
**Anterior region**						
**Delta band power**						
2-group model	0.73	1,59	0.396	2.98	1,59	0.089
3-group model	0.31	2,54	0.734	2.97	1,54	0.090
**Theta band power**						
2-group model	0.02	1,59	0.893	1.60	1,59	0.211
3-group model	0.96	2,54	0.388	1.69	1,54	0.199
**Beta band power**						
2-group model	<0.01	1,59	0.982	0.98	1,59	0.325
3-group model	0.34	2,54	0.710	0.86	1,54	0.359
**Central region**						
**Delta band power**						
2-group model	0.09	1,59	0.762	0.46	1,59	0.501
3-group model	0.16	2,54	0.853	0.44	1,54	0.511
**Theta band power**						
2-group model	0.08	1,59	0.773	0.74	1,59	0.393
3-group model	0.60	2,54	0.553	0.85	1,54	0.359
**Beta band power**						
2-group model	0.19	1,59	0.660	2.96	1,59	0.091
3-group model	0.26	2,54	0.772	2.60	1,54	0.113
**Posterior region**						
**Delta band power**						
2-group model	0.52	1,59	0.475	0.93	1,59	0.338

3-group model	0.10	2,54	0.903	0.84	1,54	0.362
**Theta band power**						
2-group model	0.03	1,59	0.853	0.43	1,59	0.514
3-group model	0.59	2,54	0.558	0.36	1,54	0.556
**Beta band power**						
2-group model	<0.01	1,59	0.976	0.42	1,59	0.518
3-group model	0.05	2,54	0.952	0.40	1,54	0.527

References

1. Garcia AN, Costa LCM, Hancock M, Costa LOP. Identifying patients with chronic low back pain who respond best to mechanical diagnosis and therapy: secondary analysis of a randomized controlled trial. *Phys Ther* (2016) 96:623–630. doi: 10.2522/ptj.20150295

2. Vriezekolk JE, Peters YAS, Steegers MAH, Davidson ENB, Van Den Ende CHM. Pain descriptors and determinants of pain sensitivity in knee osteoarthritis: a community-based cross-sectional study. *Rheumatol Adv Pract* (2022) 6:rkac016. doi: 10.1093/rap/rkac016

3. Oostenveld R, Fries P, Maris E, Schoffelen JM. FieldTrip: open source software for advanced analysis of MEG, EEG, and invasive electrophysiological data. *Comput Intell Neurosci* (2011) 2011:156869. doi: 10.1155/2011/156869
